# Chorioamnionitis accelerates granule cell and oligodendrocyte maturation in the cerebellum of preterm nonhuman primates

**DOI:** 10.1186/s12974-024-03012-y

**Published:** 2024-01-10

**Authors:** Josef Newman, Xiaoying Tong, April Tan, Toni Yeasky, Vanessa Nunes De Paiva, Pietro Presicce, Paranthaman S. Kannan, Kevin Williams, Andreas Damianos, Marione Tamase Newsam, Merline K. Benny, Shu Wu, Karen C. Young, Lisa A. Miller, Suhas G. Kallapur, Claire A. Chougnet, Alan H. Jobe, Roberta Brambilla, Augusto F. Schmidt

**Affiliations:** 1 Division of Neonatology, Department of Pediatrics, University of Miami Miller School of Medicine/Holtz Children’s Hospital, Jackson Health System, Miami, USA; 2 Division of Neonatology, Department of Pediatrics, University of California Los Angeles, Los Angeles, USA; 3 Division of Neonatology and Pulmonary Biology, Department of Pediatrics, Cincinnati Children’s Hospital Medical Center, Cincinnati, USA; 4 California National Primate Research Center, University of California, Davis, USA; 5 Division of Immunobiology, Department of Pediatrics, Cincinnati Children’s Hospital Medical Center, Cincinnati, USA; 6 The Miami Project to Cure Paralysis, Department of Neurological Surgery, University of Miami Miller School of Medicine, Miami, USA; 7 Batchelor Children’s Research Institute, 1580 NW 10Th Ave, Room 348, Miami, FL 33146 USA

**Keywords:** Chorioamnionitis, Cerebellum, Granule cell, Purkinje cell, Oligodendrocyte, Maturation

## Abstract

**Background:**

Preterm birth is often associated with chorioamnionitis and leads to increased risk of neurodevelopmental disorders, such as autism. Preterm birth can lead to cerebellar underdevelopment, but the mechanisms of disrupted cerebellar development in preterm infants are not well understood. The cerebellum is consistently affected in people with autism spectrum disorders, showing reduction of Purkinje cells, decreased cerebellar grey matter, and altered connectivity.

**Methods:**

Preterm rhesus macaque fetuses were exposed to intra-amniotic LPS (1 mg, *E. coli* O55:B5) at 127 days (80%) gestation and delivered by c-section 5 days after injections. Maternal and fetal plasma were sampled for cytokine measurements. Chorio-decidua was analyzed for immune cell populations by flow cytometry. Fetal cerebellum was sampled for histology and molecular analysis by single-nuclei RNA-sequencing (snRNA-seq) on a 10× chromium platform. snRNA-seq data were analyzed for differences in cell populations, cell-type specific gene expression, and inferred cellular communications.

**Results:**

We leveraged snRNA-seq of the cerebellum in a clinically relevant rhesus macaque model of chorioamnionitis and preterm birth, to show that chorioamnionitis leads to Purkinje cell loss and disrupted maturation of granule cells and oligodendrocytes in the fetal cerebellum at late gestation. Purkinje cell loss is accompanied by decreased sonic hedgehog signaling from Purkinje cells to granule cells, which show an accelerated maturation, and to oligodendrocytes, which show accelerated maturation from pre-oligodendrocytes into myelinating oligodendrocytes.

**Conclusion:**

These findings suggest a role of chorioamnionitis on disrupted cerebellar maturation associated with preterm birth and on the pathogenesis of neurodevelopmental disorders among preterm infants.

**Supplementary Information:**

The online version contains supplementary material available at 10.1186/s12974-024-03012-y.

## Background

The incidence of neurodevelopmental disorders is increasing among children, in part due to improved survival of at-risk newborns, such as extreme preterm infants [1, 2]. Preterm infants who are exposed to inflammation prenatally, most commonly due to inflammation of the amniotic membranes [3], or chorioamnionitis, are at particularly high risk of a wide range of neurological impairment and neurodevelopmental disorders, including cerebral palsy, attention deficit and hyperactivity disorder, cognitive impairment, and autism spectrum disorders (ASD) [4, 5]. While much of the research on brain injury and neurodevelopmental outcomes in preterm infants has focused on motor disabilities associated with supratentorial injury, disruption of cerebellar development is often seen in preterm infants, particularly underdevelopment [6, 7]. Our understanding of the cerebellar function has shifted from a largely motor and coordination role to a broader understanding of its importance in the regulation of cognitive function, including attention, memory, and executive functioning [8, 9]. In fact, the cerebellum is one of the most consistently abnormal brain areas in individuals with autism [10].

The cerebellum undergoes rapid expansion in the third trimester of gestation with peak proliferation of the granule cell precursors (GCP) in the external granule layer. These differentiate into granule cells (GC) and migrate to the internal granule layer (IGL) [11]. Insults associated with preterm birth take place during the critical time of neuronal expansion of the cerebellum. Reduction of cerebellar volume among preterm infants has been shown to persist at least into adolescence and occurs even in the absence of prior injury being detected on brain imaging [12–14]. These findings suggest that events associated with prematurity and inflammation can lead to impaired development of the cerebellum. Considering the importance of the cerebellum and of prenatal inflammation to the pathogenesis of neurodevelopmental disorders [15], such as ASD, and their association with preterm birth [16, 17], our knowledge of the effects of chorioamnionitis on the cerebellum in this critical stage of development and their potential contribution to the pathogenesis of neurodevelopmental disorders remains limited.

To fill this gap in knowledge, we performed single-nucleus RNA-sequencing (snRNA-seq) of the cerebellum in a nonhuman primate model of preterm chorioamnionitis. We demonstrate that exposure to chorioamnionitis decreases the number of Purkinje cells in the cerebellum and impairs proliferation signaling from Purkinje cells to GCs in the EGL by decreased sonic hedgehog (SHH) signaling. Chorioamnionitis also led to early maturation and myelination of oligodendrocytes in the cerebellum. These findings are consistent with histopathological findings of individuals with autism and of autopsy reports of preterm infants [10, 18, 19]. Our findings provide new insight into the mechanisms through which inflammation contributes to the disruption of brain development in preterm infants.

## Materials and methods

### Animal experiments

**Fig. 1:**
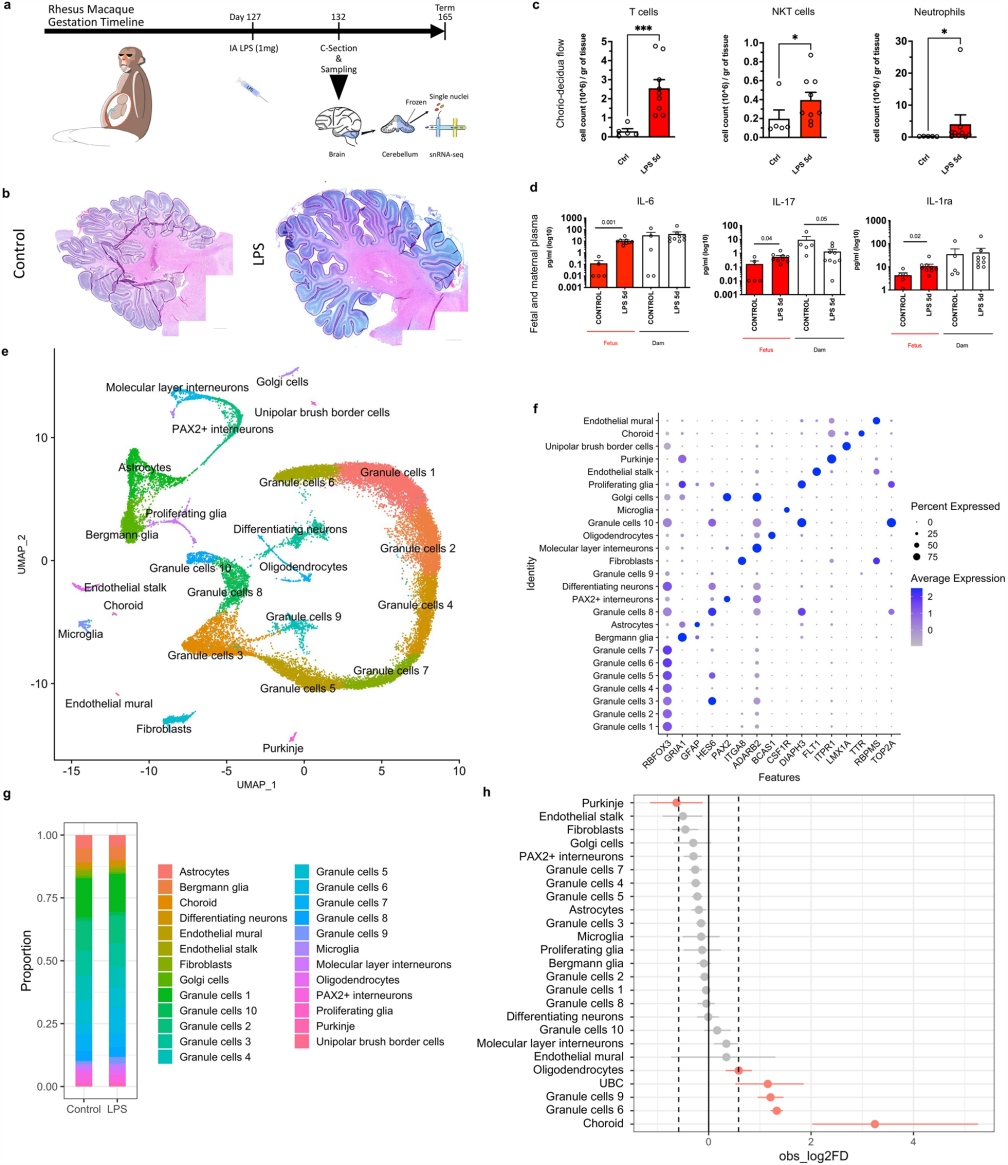
Intra-amniotic (IA) LPS induces chorio-decidual and fetal inflammation and leads to altered cerebellar cell composition. **a** Experimental design. Rhesus macaques’ fetuses at 127 days of gestation (80% gestation) received ultrasound-guided intra-amniotic injections (IA) of 1 mg of LPS from *E. coli* O55:B5 as a model of chorioamnionitis; *n* = 7/group. **b** Representative images of low power magnification of cerebellum (tile imaging, 5 ×), showing similar foliation pattern between groups. **c** Flow cytometry of the chorio-decidua cells showed increased neutrophils, T cells and NKT cells at 5 days in LPS animals (*n* = 5/group). **d** Multiplex ELISA for cytokines in the maternal and fetal plasma from controls and LPS animals shows increased IL-6 as well as increased IL-17 and IL-1ra in the fetal plasma, with maternal increase in IL-17 only. (*n* = 5/group). **e** UMAP visualization of single-nuclei RNA-seq from 30,711 cerebellar nuclei. Clustering using the Seurat package revealed 24 unique cell clusters in the developing cerebellum; *n* = 2/group. **f** Dotplot of top cell type markers based on the top differentially expressed genes in each cluster and on cell-specific and cell-enriched markers reported in the literature. **g** Proportion of each identified cell cluster by condition showing predominance of GCs in control and LPS animals. **h** Differential proportion of cell populations in each cluster between control and LPS-exposed fetuses by permutation test. LPS decreased number of Purkinje cells and increased number of oligodendrocytes, unipolar brush border cells (UBC), and GCP/GC clusters 9 and 6

Animal protocols were reviewed and approved by the institutional IACUC at the University of California Davis. Time-mated pregnant rhesus macaques at 127 days of gestation (80% of term gestation) were treated with intra-amniotic (IA) lipopolysaccharide (LPS) 1 mg (*E*. *coli* O55:B5, Sigma-Aldrich) diluted in 1 ml of sterile saline (LPS). Controls received no intervention. Animals were delivered *en cul* by cesarean section at 5 days after intra-amniotic LPS (Fig. 1a). No spontaneous labor or fetal losses were observed in the intervention or control groups. Fetuses were humanely euthanized with pentobarbital. After euthanasia, the cerebellum was dissected, and the hemispheres were separated along the midline of the vermis. The left hemisphere with the left side of the vermis was frozen for molecular analysis and the right hemisphere was fixed in 10% formalin and embedded in paraffin for histological analyses. Data of animals included in the study are shown in Table 1.

**Table 1:** Characteristics of animals included in the study

	Control (*n* = 8)	IA LPS (*n* = 9)
Gestational age at delivery (days median, range)	133 (130–135)	133 (131–134)
Birth weight (g mean, SD)	330 ± 35	321 ± 37
Gender (F/M)	2/6	4/5
Amniotic fluid cell count (10^4^/ml median, range)	0 (0–40)	30 (22–60)*
Maternal plasma WBC (10^9^/l average, SD)	8.2 ± 2.3	5.6 ± 1.7*
Maternal plasma neutrophils (% average, SD)	78 ± 6	51 ± 16*
Maternal plasma lymphocytes (% average, SD)	18 ± 5	43 ± 15*
Maternal plasma platelets (10^9^/l average, SD)	356 ± 71	349 ± 88
Fetal plasma WBC (10^9^/l average, SD)	2.4 ± 0.6	3.3 ± 0.8*
Fetal plasma neutrophils (% average, SD)	9.7 ± 6	23 ± 5*
Fetal plasma lymphocytes (% average, SD)	85 ± 8	73 ± 6*
Fetal plasma platelets (10^9^/l average, SD)	369 ± 72	314 ± 91*

*WBC* white blood cell count, *SD* standard deviation

*p < 0.05

### Chorion–amnion–decidua dissection and flow cytometry of chorio-decidua cells

At delivery, extra-placental membranes were collected and dissected away from the placenta as previously described [20] . The cells of the chorio-decidua were scraped and the amnion and remaining chorion tissue were separated with a forceps. The chorio-decidua cells were washed and digested with Dispase II (Life Technologies, Gran Island, NY, USA) plus collagenase A (Roche, Indianapolis, IN, USA) followed by DNase I (Roche) treatment. Cells suspensions were filtered, red blood cells lysed, and the suspension was prepared for flow cytometry. Cell viability was > 90% by trypan blue exclusion test. Multiparameter flow cytometry and gating strategy of the difference leukocyte subpopulations was done as previously described [20]. Monoclonal antibodies used are listed in Table 2. Cells were treated with 20 μg/ml of human immunoglobulin G (IgG) to block Fc receptors, stained for surface markers for 30 min at 4 °C in PBS, washed, and fixed in fixative stabilizing buffer (BD Bioscience). All antibodies were titrated for optimal detection of positive populations and similar mean fluorescence intensity. At least 500,000 events were recorded for each sample. Doublets were excluded based on forward scatter properties, and dead cells were excluded using LIVE/DEAD Fixable Aqua dead cell stain (Life Technologies). Unstained and negative biological population were used to determine positive staining for each marker. Data were analyzed using FlowJo version 9.5.2 software (TreeStar Inc., Ashland, OR, USA).

**Table 2:** Antibodies used in flow cytometry experiments

Antibody	Clone	Conjugation	Manufacturer
CD45	D058-1283	PE-CF594	BDBioscience
HLA-DR	L243	Brilliant Violet 570 PercPCy5.5	Biolegend
CD3	SP34-2	APC-Cy7	BDBioscience
CD14	TUK4	Pacific Blue	Thermofisher
CD56	NCAM16.2	PE-Cy7	BDBioscience
CD88	P12/1	Alexa Fluor 647	AbD Serotec
CD19	HIB19	Alexa Fluor 700	Biolegend
CD20	2H7	Alexa Fluor 700	Biolegend
CD16	3G8	Alexa Fluor 700	BDBioscience
CD63	H5C6	Pacific Blue	Biolegend
Live/Dead		Aqua	BDBioscience

### Cytokine concentration measurement

We measured cytokine concentration in maternal and fetal plasma by Luminex technology using multiplex kits for nonhuman primate (Millipore, Burlington, MA, USA). Each 96-well filter plate was blocked with 100 μl of blocking buffer for 30 min, followed by vacuum filtration at 2 psi. The 25 × bead mix was vortexed for 1 min and sonicated for 30 s. prior to diluting. The bead mix was diluted in wash buffer, and 50 μl added to each well followed by vacuum filtration. The standard was dissolved in the supplied medium. Samples and standards were added to the plates in triplicate (plasma samples were diluted 1:2). The plates were placed on a shaker at 4 °C overnight. The following day, the medium was vacuum-filtered, and 50 μl of detection antibody was added to each well. The wells were washed four times with 100 μl wash buffer and the plates were incubated on a shaker at room temperature for 1 h in the dark. The plates were washed again four times with 100 μl wash buffer. Streptavidin-PE (50 μl) was added to each well and the plate was placed on a shaker for 15 min at room temperature in the dark. The wells were washed four times with 100 μl wash buffer and the beads were resuspended in 150 μl wash buffer for analysis. Immediately prior to analysis, the plates were shaken to resuspend the beads. Plates were read on a Luminex 100 (Luminex Corporation, Austin, TX, USA).

### Immunofluorescence and immunohistochemistry

Paraffin-embedded tissue sections underwent heat-assisted antigen retrieval with citrate buffer (pH 6.0). For immunohistochemistry endogenous peroxidase activity was reduced with H_2_O_2_ treatment. Nonspecific binding sites were blocked with 4% bovine serum diluted in PBS followed by incubation with primary antibodies overnight at 4 °C (Table 3). The following day, tissue sections were incubated with the appropriate species-specific biotinylated or Alexa Fluor-conjugated secondary antibody diluted in 1:200 in 4% bovine serum for 2 h at room temperature. For immunohistochemistry with DAB, tissue sections incubated with biotinylated secondary antibody were washed and antigen/antibody complexes were visualized using Vectastain ABS peroxidase kit (Vector Laboratories, Burlingame, CA, USA) followed by counterstaining with Harris hematoxylin. For immunofluorescence, sections incubated with Alexa Fluor-conjugated secondary antibody were washed and mounted with VectaShield Hardset Mounting Media (Vector Laboratories). Histological analyses and cell counts were performed in the entire cerebellum.

**Table 3:** Antibodies used in flow immunohistochemistry and Western blots

Antibody	Host	Application	Dilution	Catalog	Manufacturer
Beta-actin	Mouse	WB	1:10,000	A5441	Sigma-Aldrich
Calbindin	Rabbit MAb	IF	1:200	Ab108404	Abcam
MBP	Mouse MAb	IF	1:3000	ab62631	Abcam
MBP	Mouse MAb	WB	1:10,000	ab62631	Abcam
SHH	Mouse MAb IgG1	IHC	1:50	ab135240	Abcam
SHH	Mouse MAb IgG1	WB	1:1000	ab135240	Abcam
Anti-mouse IgG	Goat	WB	1:10,000	31,430	ThermoFisher
Anti-rabbit AF488	Goat	IF	1:200	A32731	ThermoFisher
Anti-rabbit IgG	Goat	WB	1:10,000	31,460	ThermoFisher
Anti-rabbit IgG	Goat	IHC	1:200	BA1000	Vector Labs

*IHC* immunohistochemistry, *IF* immunofluorescence, *MAb* monoclonal antibody, *MBP* myelin basic protein, *SHH* sonic hedgehog, *WB* Western blotting

### Western blot analyses

Protein concentrations of tissue homogenates or EVs were measured by BCA protein assay using a commercial kit (Pierce Biotechnology Inc., Rockford, IL, USA). Total proteins (20 µg/sample) were fractionated by SDS-PAGE on 4–12% Tris–glycine precast gradient gels (ThermoFisher, Waltham, MA, USA) and then transferred to nitrocellulose membranes (Amersham, Piscataway, NJ, USA). The membranes were incubated overnight at 4 °C with the respective primary antibodies and then incubated for 1 h at room temperature with HRP-conjugated secondary antibodies. Antibody bound proteins were detected using ECL chemiluminescence methodology (Amersham, Piscataway, NJ, USA). The intensities of protein bands were quantified by ImageJ [16]. Band density was then corrected for beta-actin band density in the same lane. Values were then divided for the average control value to represent fold change relative to control.

### Statistical analyses

GraphPad Prism (GraphPad Software, La Jolla, CA, USA) was used to graph and analyze data. Statistical differences between groups were analyzed using Mann–Whitney *U*-tests for cytokine concentration measurements and cellular composition from flow cytometry results. Results were considered significantly different for *p* values ≤ 0.05. However, due to the limited number of samples per group, we also report trends (*p* values between 0.05 and 0.1).

### Single-nucleus RNA isolation, sequencing, and mapping

Nuclei isolation and single-nuclei RNA sequencing (sn-RNAseq) were performed by Singulomics Corporation (Singulomics.com). Flash frozen cerebellar hemispheres and half of the cerebellar vermis from 2 rhesus macaque fetuses per group were homogenized and lysed with Triton X-100 in RNase-free water for nuclei isolation. Isolated nuclei were purified, centrifuged, and resuspended in PBS with RNase inhibitor, and diluted to 700 nuclei/µl for standardized 10 × capture and library preparation protocol using 10 × Genomics Chromium Next GEM 3’ Single Cell Reagent kits v3.1 (10 × Genomics, Pleasanton, CA, USA). The libraries were sequenced with an Illumina NovaSeq 6000 (Illumina, San Diego, CA, USA). Raw sequencing files were processed with CellRanger 5.0 (10 × Genomics) and mapped to the rhesus macaque genome (Mmul_8.0.1) with the option to include introns and generated count matrices.

### SnRNA-seq processing, clustering, and differential expression

Downstream analyses were performed on the Seurat package version 3.1.0 for R [21]. Each sample was processed separately and then integrated for cell type identification and comparison analyses. After importing the CellRanger data using the Read10x function and creating a Seurat object for each sample, samples were normalized, scaled, and the top 2000 variable features were identified followed by nuclei clustering. After clustering, ambient RNA was removed with SoupX [22]. Cleaned samples were then filtered removing nuclei with < 500 genes or > 5% mitochondrial RNA. The number of nuclei and genes per sample is shown in Table 4. After filtering, samples were integrated using the FindIntegrationAnchors function with the parameter dims = 1:30 followed by IntegrateData function with dims = 1:30. The integrated data were scaled, principal component analysis (PCA) was performed with npcs = 30, and nuclei were clustered with resolution = 0.5. Conserved cell types for each cluster were identified using the FindAllMarkers function with default parameters. Cell types were identified based on known cell markers and previously published datasets of cerebellum single-cell sequencing [23–25]. Differences in the proportion of cells in clusters between control and IA LPS was performed using the permutation test on the scProportionTest package for R [26]. Global and cluster subset differential expression analyses between Control and IA LPS were performed using the FindMarkers function with default parameters. Overrepresentation analysis of differentially expressed genes was performed for Gene Ontology terms and KEGG Pathways on ToppCluster [27] and using the DEenrichRPlot function on Seurat [28, 29].

**Table 4:** Characteristics of samples included in snRNA-seq

	Control 1	Control 2	LPS 1	LPS 2
Gestational age at delivery	130	130	131	133
Birth weight (g)	290	290	282	348
Gender	F	M	F	M
Number of nuclei	9021	8593	7002	6095
Mean reads per nuclei	25,192	30,437	24,008	45,124
Median genes per nuclei	2118	1203	1990	1589

### Pseudotime analyses

To determine lineage trajectories and determine differences across conditions, we performed pseudotime analyses with Monocle3 [30–32] and Slingshot [33] followed by *condiments* [34]. For analysis on Monocle3 [30], *celldataset* objects were created from the individual Seurat objects and combined using the combine_cds function and preprocessed. We then performed dimensionality reduction using UMAP [35] followed by cell clustering. Specific cell types of interest were subset, clustered and a principal graph was fit within each partition using the learn_graph function. Cells were then ordered in pseudotime using the plot_cells function. For analysis with Slingshot [33] a Seurat object for each condition was created and converted to a *single cell experiment* object followed by conversion to slingshot object using the slingshot function with reducedDim = “PCA”. Objects were then filtered and normalized following the standard protocol. We then performed the topologyTest on condiments [34] to determine differences in lineage trajectories between conditions.

### Cell communication analyses

Cell–cell interactions were inferred with CellChat [36] based on known ligand-receptor pairs in difference cell types. To identify perturbed cell–cell communication networks in chorioamnionitis we loaded the condition specific Seurat objects into cell chat using the createCellChat function, followed by the preprocessing functions indentifyOverEpressedGenes, identifyOverExpressedinteractions, and projectData with default settings. For comparison between Control and IA LPS, we applied the computeCommunProb, computeCommunProbPathway, and aggregateNet functions using standard parameters and fixed randomization seeds. To determine signal senders and receivers we used the netAnalysis_signalingRole function on the netP data slot.

## Results

### Intra-amniotic LPS induces persistent fetal inflammation after 5 days

To model chorioamnionitis in a preterm nonhuman primate we performed ultrasound-guided IA injection of 1 mg of LPS (*E. coli* O55:B5) on gestational day 127 (80% gestation, term is 165 days) (Fig. 1a, Table 1). This model has been previously shown to induce inflammation at the maternal–fetal interface, fetal systemic inflammatory response, and fetal neuroinflammation up to 48 h after injection [37, 38]. Flow cytometry of the chorio-decidua showed that, at 5 days after IA LPS injection, there was a predominantly neutrophilic infiltrate in the chorio-decidua (Fig. 1c, Additional file 1: Fig. S1). Inflammatory infiltrate in the membranes was associated with elevation of IL-6, IL-17, and IL-1ra in the fetal plasma by multiplex ELISA, without elevations of cytokines in the maternal plasma at 5 days (Additional file 1: Fig. S1d, Additional file 2: Fig. S2). There was no alteration in foliation pattern between groups (Additional file 1: Fig. S1b).

### snRNA-seq reveals major developing cell types in the cerebellum at late gestation

To determine the effects of chorioamnionitis on the developing cerebellum we used unbiased high-throughput snRNA-seq to examine transcriptional populations of the cerebellum from preterm rhesus macaque fetuses exposed to IA LPS or placebo. We then analyzed the transcriptome from 30,711 single nuclei (17,614 nuclei from 2 controls, 13,097 nuclei from 2 chorioamnionitis) to an average depth of 25,192 to 45,124 reads per nuclei (Table 2). Nuclei were clustered based on their expression profile, and we identified 24 cell clusters that were annotated based on published cell markers [23–25] (Fig. 1e, f; Additional file 6: Table S1). We then analyzed the cell type composition in the cerebellum of controls and LPS-exposed fetuses (Fig. 1g). Permutation test [26] demonstrated a decrease in the proportion of Purkinje cells and an increase in oligodendrocytes, unipolar brush border cells (UBC), choroid cells, and GCs clusters 9 and 6 in LPS-exposed fetuses (Fig. 1h), showing that chorioamnionitis alters the cell composition of the fetal cerebellum.

### Chorioamnionitis accelerates cerebellar GC maturation

**Fig. 2:**
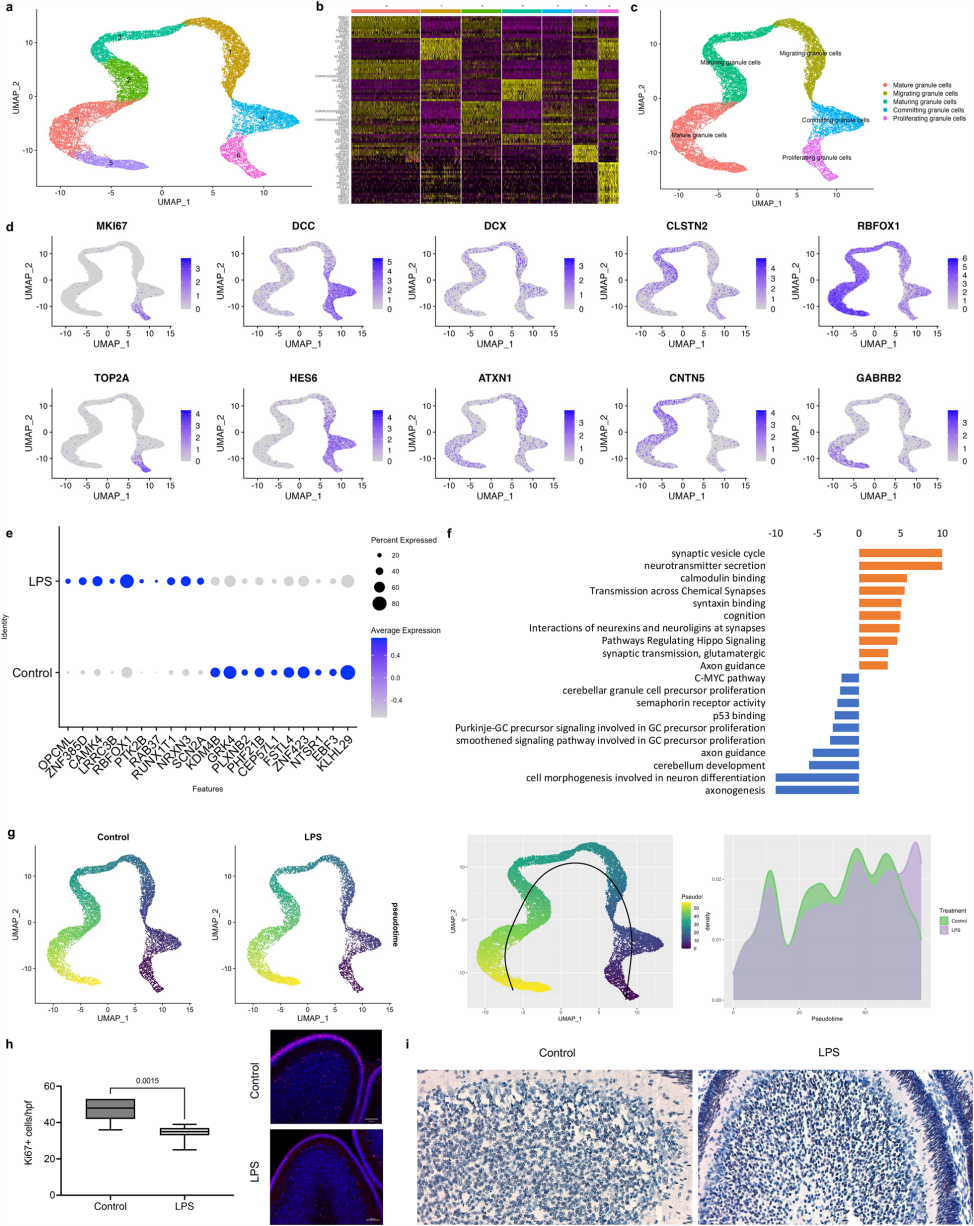
Prenatal inflammation disrupts cerebellar GCP/GC development increasing the number of mature GCs.** a** UMAP plot of re-clustered GCPs/GCs identified 7 distinct populations of GCPs/GCs in the developing cerebellum. **b** Heatmap of the top 10 differentially expressed genes in each GCP/GC cluster identified shows similarities between some of the clusters which were regrouped for classification based on gene expression patterns. **c** Regrouped clusters based on gene expression patterns shows the presence of GCPs/GCs at different developmental stages from proliferating to mature GCs. **d** Feature plots of identified cell markers for proliferative GCPs (MKI67 and TOP2A), GCPs/GCs exiting cell cycle and committing to differentiation (DCC and HES6), migrating GCPs (DCX and ATXN1), GCs with increased mature cell markers and markers that the GCs have exited the EGL (CNTN2 and CLSTN1), and mature GCs (RBFOX3 and GABRB2). **e** Dotplot of top genes differentially expressed in LPS-exposed fetuses vs. control in all clusters of the cerebellar GCPs/GCs. **f** Overrepresentation analysis of differentially expressed genes in LPS-exposed fetuses vs controls. Genes induced by LPS were associated with biological processes for synapse function and hippo signaling pathway. Genes suppressed by LPS were associated with Purkinje-GCP signaling involved in GCP proliferation, cerebellum development, and cell morphogenesis involved in neuron differentiation. **g** Pseudotime analysis on monocle3 and slingshot using the condiments package to identify differences in proportion of cells along pseudotime shows increased GC maturation LPS-exposed fetuses. **h** Number of Ki67 + cells per high power field (hpf) in the cortex of LPS-exposed compared to control animals, showing decreased cell proliferation, counts done on high power (20x) and low power shown in picture (5 ×). **i** Neurod1 staining in the cerebellum showing increased number of Neurod + cells in the cerebellar cortex in LPS-exposed fetuses (20 ×)

Cerebellar GCs are the most abundant neurons in the brain and were the most abundant cell type identified in our dataset. Given the increased proportion of two GC clusters in LPS-exposed fetuses, we re-clustered the GC populations for further analyses. Re-clustering of the GC identified seven distinct clusters based on their expression profile (Fig. 2a, b). Analysis of the top cluster gene markers and established markers for neurons at different developmental stages identified GCs at five stages of development in our dataset: proliferating GCPs, committing GCs, migrating GCs, maturing GCs, and mature GCs (Fig. 2c, d, Additional file 7: Table S2). Differential expression analysis of gene expression between LPS-exposed and control cerebellum identified 317 differentially expressed genes using a threshold of log fold-change > 0.25 and FDR < 0.1 (Fig. 2e, Additional file 8: Table S3). Overrepresentation analysis of differentially expressed genes was performed on ToppCluster [27] for genes induced and suppressed in chorioamnionitis. Genes induced in GCs of LPS-exposed fetuses were associated with synapse transmission and hippo signaling pathways, while suppressed genes were associated with cerebellum development, and Purkinje cell–GCP signaling involved in GCP proliferation (Fig. 2f). To further explore the effect of chorioamnionitis on GCP proliferation and GC maturation, we performed pseudotime analyses on Monocle3 [30] and Slingshot [33] followed by differential topology between conditions[34] (Fig. 2g). Both pseudotime analyses methods showed an increased proportion of the most mature GCs, suggesting that chorioamnionitis accelerates cerebellar GC maturation.

### Chorioamnionitis decreases the relative abundance of Purkinje cells in the developing cerebellum

**Fig. 3:**
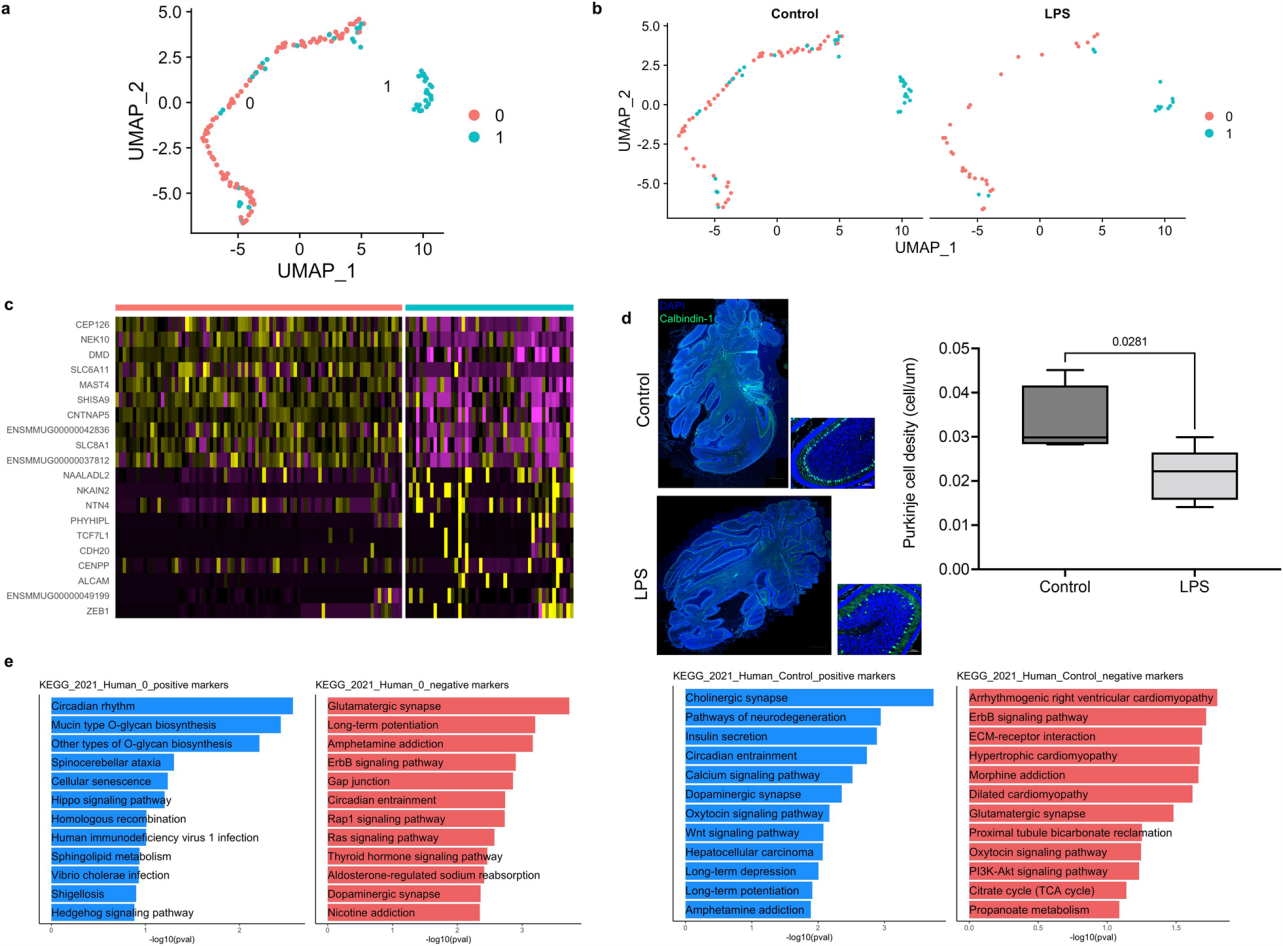
Prenatal inflammation decreases Purkinje cell density in the cerebellum. **a** UMAP of the cell subset identified as Purkinje cells. Re-clustering of Purkinje cells identified 2 subpopulations. **b** UMAP of Purkinje cells by condition. **c** Heatmap of top differentially expressed genes in each Purkinje cell cluster. **d** Immunofluorescence (magnification 20X) for Calbindin-1 and Purkinje cell density analysis showing decreased density of Purkinje cells in LPS-exposed fetuses (*n* = 6 animals/group). **e** Gene set enrichment analysis for KEGG Pathways of genes differentially expressed in cluster 1 compared to cluster 0 of Purkinje cells

Since IA LPS decreased the proportion of Purkinje cells, we sought to further determine the effect of chorioamnionitis on Purkinje cell numbers and gene expression. Sub-setting and re-clustering of the Purkinje cells revealed 2 clusters (Fig. 3a) with distinct cell markers (Fig. 3b, Additional file 6: Table S1). Overrepresentation analysis for KEGG Pathways of genes differentially expressed in cluster 1 compared to cluster 0 of Purkinje cells showed that genes in cluster 0 were associated with Hippo signaling, cellular senescence, and SHH signaling while genes in cluster 1 were associated with glutamatergic synapse, Rap1 signaling pathway, and Ras signaling pathway, suggesting distinct developmental functions of these subpopulations (Fig. 3c). Differential gene expression of genes expressed in Purkinje cells in LPS-exposed compared to control fetuses resulted in 302 regulated genes (Fig. 3d, Additional file 9: Table S4). Pathway overrepresentation analysis of genes differentially expressed in LPS-exposed fetuses compared to control showed that genes suppressed by chorioamnionitis were associated with cholinergic synapse, dopaminergic synapse, and Wnt signaling, and genes induced by LPS were associated with ErbB signaling pathway and glutamatergic synapse suggesting that LPS suppressed developmental pathways and synapse formation in Purkinje cells (Fig. 3e). We then performed immunofluorescence for calbindin-1 and measured the Purkinje cell linear density by dividing the number of Purkinje cells by the length of the Purkinje cell folia in 3 cerebellar folia per animal [39]. There was a nonsignificant trend towards decreased Purkinje cell linear density in LPS-exposed fetuses consistent with the finding of decrease proportion of Purkinje cells in the single-nuclei analysis (Fig. 3e).

### Chorioamnionitis accelerates oligodendrocyte maturation in the cerebellum

**Fig. 4:**
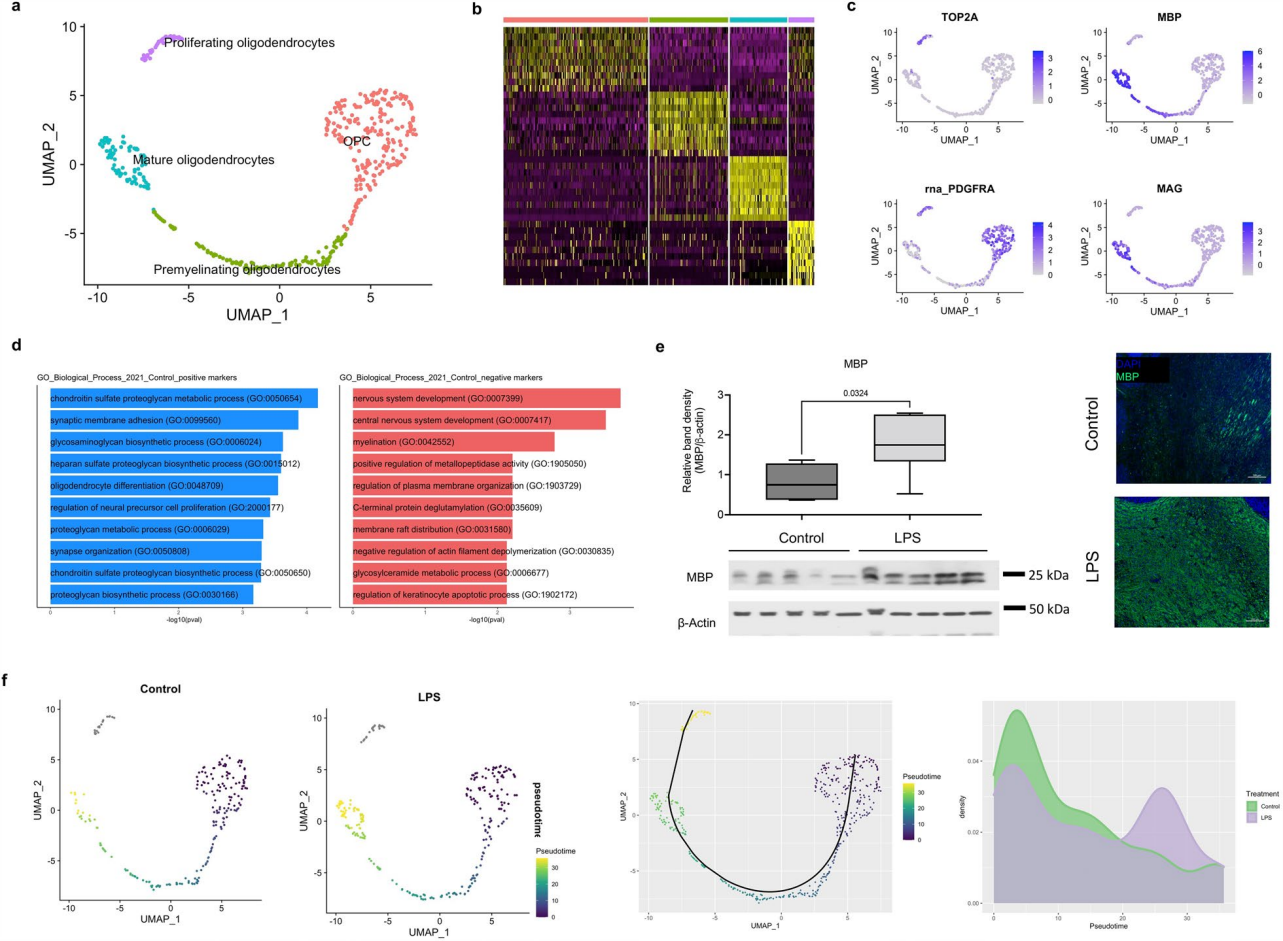
Prenatal inflammation disrupts oligodendrocyte development with increased expression of myelination-associated genes. **a** UMAP plot of the oligodendrocyte cluster. Re-clustering showed 4 subpopulations of oligodendrocytes that were identified as stages of oligodendrocyte development based on gene expression. **b** Heatmap of top differentially expressed gene in each oligodendrocyte cell cluster. **c** Expression of markers of oligodendrocyte development in proliferating oligodendrocytes (TOP2A), oligodendrocyte precursor cells (OPC) (PDGFRA), and myelinating/mature oligodendrocytes (MBP and MAG). **d** Gene set enrichment analysis for biological processes of genes differentially expressed in the oligodendrocyte cluster in LPS compared to control fetuses with blue as upregulated in control and red as downregulated in control. **e** Western blot and immunofluorescence for myelin basic protein (MBP) in the fetal cerebellum, *p* value = 0.03 (*n* = 5 animals/group). **f** Pseudotime analysis on monocle3 and slingshot using the condiments package to identify differences in proportion of cells along pseudotime shows accelerated oligodendrocyte maturation LPS-exposed fetuses

Given the increased proportion of oligodendrocytes in LPS-exposed fetuses relative to control on the single-nuclei data, we sought to further determine the effect of chorioamnionitis on oligodendrocyte proliferation and maturation. Sub-setting and re-clustering of oligodendrocytes revealed four distinct clusters (Fig. 4a, b) which were identified based on the top cell markers (Additional file 6: Table S1) and genes known to be differentially expressed during oligodendrocyte maturation, specifically: topoisomerase IIα (TOP2A) in proliferating oligodendrocytes, platelet-derived growth factor receptor α (PDGFRA) in oligodendrocyte progenitor cell (OPC), and myelin basic protein (MBP) and myelin-associated glycoprotein (MAG) in premyelinating oligodendrocytes and mature oligodendrocytes (Fig. 4a, c). We then performed differential gene expression analysis in the oligodendrocyte cluster of LPS compared to control fetuses. Overrepresentation analysis of genes differentially expressed between LPS and control revealed that genes suppressed by LPS were associated with oligodendrocyte differentiation and genes induced by chorioamnionitis were associated with myelination (Fig. 4d, Additional file 10: Table S5). We then analyzed cerebellar myelination by Western blotting for MBP, which showed increased MBP in the cerebellum of LPS-exposed fetuses (Fig. 4e). To further validate those findings we performed pseudotime analysis on Monocle3 [30] and Slingshot [33] followed by differential topology between conditions [34]. On both analyses we observed an increased proportion of mature oligodendrocytes in the chorioamnionitis animals (Fig. 4f).

### Chorioamnionitis induces formation of a new cluster of UBCs

**Fig. 5:**
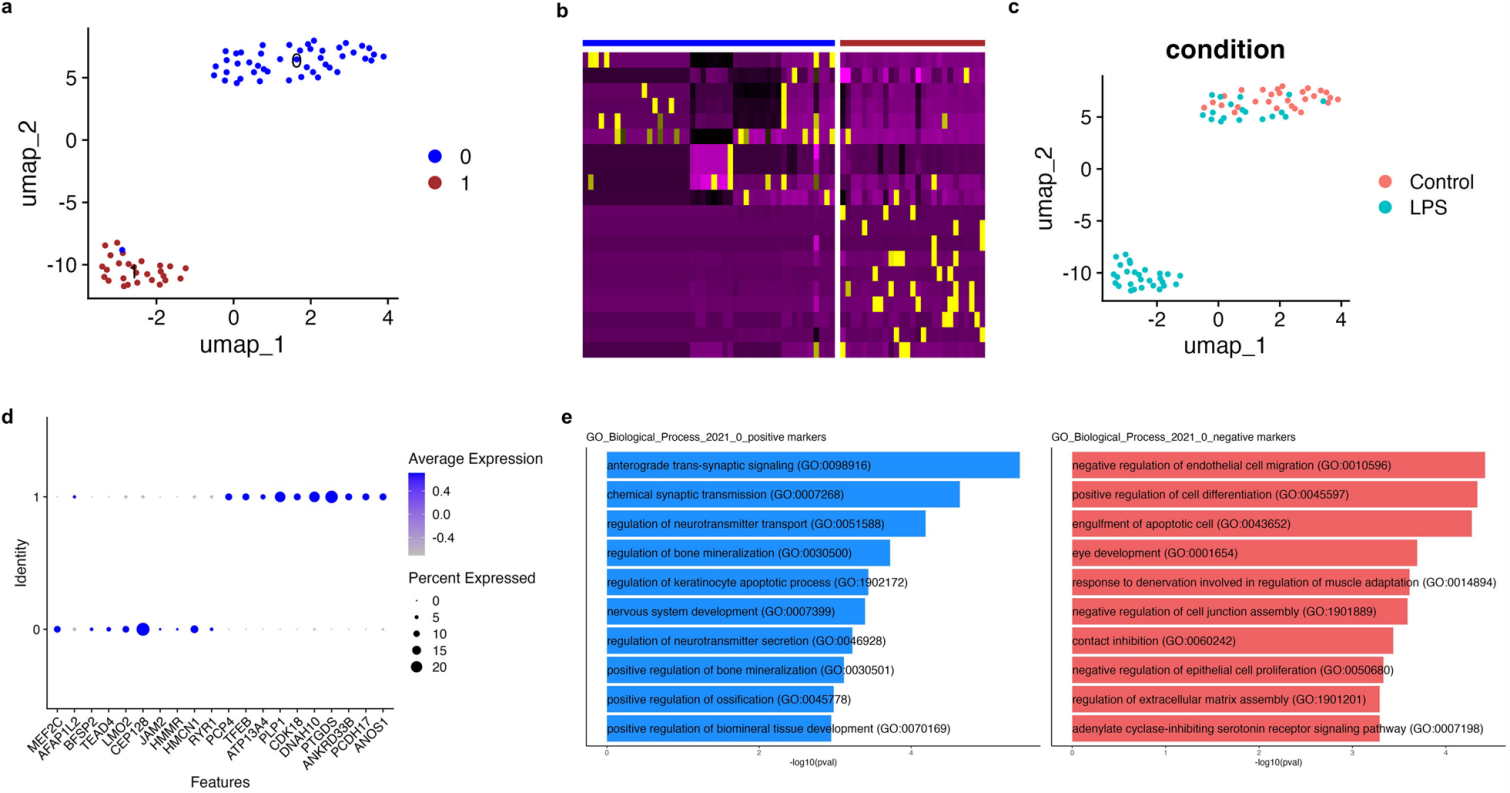
Chorioamnionitis disrupts UBC homeostasis. **a** UMAP plot of the UBC cluster. Re-clustering showed 2 subpopulations of UBCs. **b** Heatmap of top differentially expressed genes in each UBC cluster. **c** Distribution of cells in each cluster by condition showing that cells in cluster 1 comprised exclusively UBCs from LPS-exposed fetuses. **d** Gene set enrichment analysis for biological processes of genes differentially expressed in the UBC cluster 1 compared to cluster 0 showing changes of genes associated with synaptic signaling and cell junction

Following the initial analysis demonstrating an increase in UBCs induced by chorioamnionitis, we performed a subset analysis of these cells. Clustering of UBCs revealed two clusters (Fig. 5a), with top identified based on specific cell markers differentially expressed between clusters (Fig. 5b, Additional file 6: Table S1). Interestingly, while cluster 0 was comprised both from controls and LPS-exposed fetuses, cluster 1 only had cells from LPS-exposed fetuses (Fig. 5c). Overrepresentation analysis of genes differentially expressed between the two UBS clusters (Fig. 5d) showed that genes differentially expressed in cluster 0 were positively associated with anterograde trans-synaptic signaling, chemical synaptic transmission, and regulation of neurotransmitter secretion (Fig. 5e, left), and negatively associated with cell differentiation, engulfment of apoptotic cell, and contact inhibition (Fig. 5e, right). Considering the observed reduction in Purkinje cells in LPS-exposed animals, UBCs in those animals would have decreased synaptic connections and cell–cell contact which could explain the suppression of terms associated with neurotransmission and induction of genes associated with contact inhibition and negative regulation of cell junction in cluster 1 comprised LPS-exposed fetuses only compared to cluster 0. These findings, however, do not explain the increase in UBC as we did not identify signaling.

### sn-RNAseq reveals microglial and astrocyte diversity in the developing cerebellum

To determine the effect of chorioamnionitis on microglial and astrocyte activation in the cerebellum, we individually analyzed the microglia and astrocyte clusters. We found 3 distinct microglia clusters based on expression profile (Additional file 3: Fig. S3a, b). There was no difference in cluster distribution between control and LPS (Additional file 3: Fig. S3c). Top cell markers for microglial clusters (Additional file 6: Table S1) revealed canonical microglia markers triggering receptor expressed on myeloid cells 2 (TREM2) and C–X3–C Motif Chemokine Receptor (CX3CR1) and cluster-specific microglial markers, Plexin domain containing 2 (PLXDC2), disable 2 (DAB2), and cell adhesion molecule 2 (CADM2) (Additional file 3: Fig. S3d). Overrepresentation analysis of the top differentially expressed genes in each cluster revealed specific microglial functions. Genes differentially regulated in cluster 0 were associated with mononuclear cell differentiation, microglia migration, and regulation of cell adhesion (Additional file 3: Fig. S3e), cluster 1 was associated with ERK1 and ERK2 cascade, and receptor mediated endocytosis (Additional file 3: Fig. S3f), and cluster 2 was associated with cell adhesion, channel activity, and synapse organization (Additional file 3: Fig. S3g), suggesting cluster-specific microglia roles in the developing cerebellum.

Sub-setting and re-clustering of astrocytes revealed 4 distinct cell clusters based on gene expression pattern (Additional file 4: Fig. S4a, b) that showed similar distribution between control and LPS-exposed fetuses (Additional file 4: Fig. S4c). Top cell cluster markers for the four astrocyte clusters identified revealed cluster-specific and cluster enriched gene markers (Additional file 4: Fig. S4d; Additional file 6: Table S1), which were associated with specific astrocyte biological functions on overrepresentation analysis. Cluster 0 was associated with epidermal growth factor signaling which is crucial for astrocyte transition from quiescent to activated state (Additional file 4: Fig. S4e). Cluster 1 was associated with inositol lipid-mediated signaling, regulation of gene expression, and endothelial proliferation (Additional file 4: Fig. S4f). Cluster 2 was associated with extracellular matrix organization, regulation of synapse potentiation, and axonogenesis (Additional file 4: Fig. S4g). Cluster 3 was associated with synaptic transmission and signaling, dendrite self-avoidance, and glutamate signaling (Additional file 4: Fig. S4h). These findings support the presence of functionally diverse population of astrocytes in the late gestation cerebellum.

### Cell–cell communication in the cerebellum is disturbed by chorioamnionitis

**Fig. 6:**
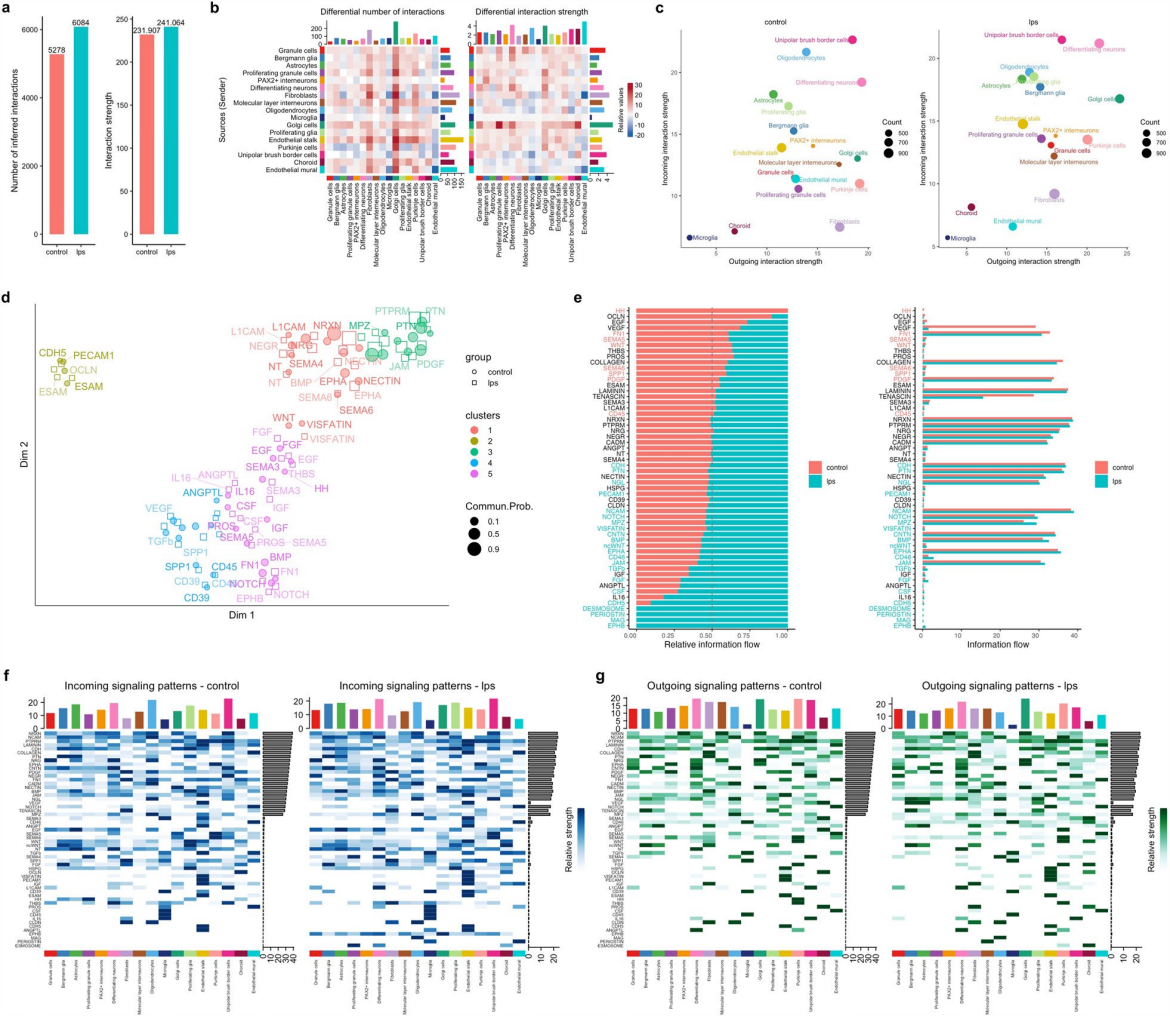
Cell–cell communication is disrupted by antenatal inflammation. **a** Global number of inferred interactions (left) and global strength of interaction (right) in the cerebellum of control and LPS-exposed fetuses. **b** Differential number of interactions and interactions strength between each cell type identified on clustering. Red represents increased, blue represents decreased. The top colored barplot represents the sum of column values displayed in the heatmap (incoming interactions), the right colored barplot represents the sum of row values (outgoing interactions). **c** Scatterplot of outgoing and incoming interaction strength for each cell cluster in two dimensions. **d** Scatterplot and classification of communication networks based on their functional similarity. **e** Bar plots of relative (right) and absolute information flow showing conserved and context-specific signaling pathways in control and LPS. **f** Heatmap of incoming signaling patterns in each cluster in control (right) and LPS (left). **g** Heatmap of incoming signaling patterns in each cluster in control (right) and LPS (left)

Initial gene differential expression analysis suggested suppression of Purkinje cell–GCP signaling. To further determine the effect of chorioamnionitis on the intercellular communication networks in the developing cerebellum, we analyzed our data on CellChat [36]. CellChat uses established ligand-receptor knowledge for quantitative inference and quantitation of intercellular communication networks. Quantification of the global number of interactions and interaction strength among the cell clusters identified showed that LPS increased the number and strength of inferred interactions among the cell clusters (Fig. 6a, b). LPS particularly decreased the incoming interaction strength for oligodendrocytes, Purkinje cells, and endothelial mural cells, and increased the incoming interaction strength for proliferating GCs, Bergman glia, and Golgi cells (Fig. 6c). Moreover, LPS decreased the number and strength of interaction between UBCs and Purkinje cells (Fig. 6b).

We then investigated how the cell–cell communication architecture changed in chorioamnionitis by projecting the inferred cell communication networks from control and LPS in a shared two-dimensional space based on their functional similarity (Fig. 6d). We identified 5 major signaling groups based on functional similarity of which some were unique to control such as Hedgehog (HH, cluster 5), and others unique to LPS such as EphrinB (EPHB) and Desmosome in cluster 5, and periostin and MAG in cluster 4. In addition, other pathways such as WNT, bone morphogenetic protein (BMP), and netrin-F ligand (NGL) were classified into different groups in control and LPS, suggesting that these pathways changed their cell–cell communication architecture in chorioamnionitis (Fig. 6d, Additional file 5: Fig. S5).

Next, we determined the conserved and context-specific signaling pathways in control and LPS by comparing the information flow for each signaling pathway (Fig. 6e). We identified HH as a control-specific pathway, while desmosome, periostin, MAG, and EPHB as LPS-specific pathways, albeit with small absolute information flow. Moreover, we identified significant differences in information with both increases in LPS (such as WNT and PDGF) as well as decreases in LPS (such as NOTCH, BMP, and FGF) relative to control. We then analyzed the cell-specific incoming and outgoing signaling patterns for the pathways identified above (Fig. 6f, g). Purkinje cells were the unique source of HH signaling in controls targeting multiple cell types and HH signaling was not present in LPS. Oligodendrocytes were the unique source and target of MAG in LPS, and MAG signaling was not present in controls.

### Chorioamnionitis impairs SHH signaling from Purkinje cells to proliferating GCs

**Fig. 7:**
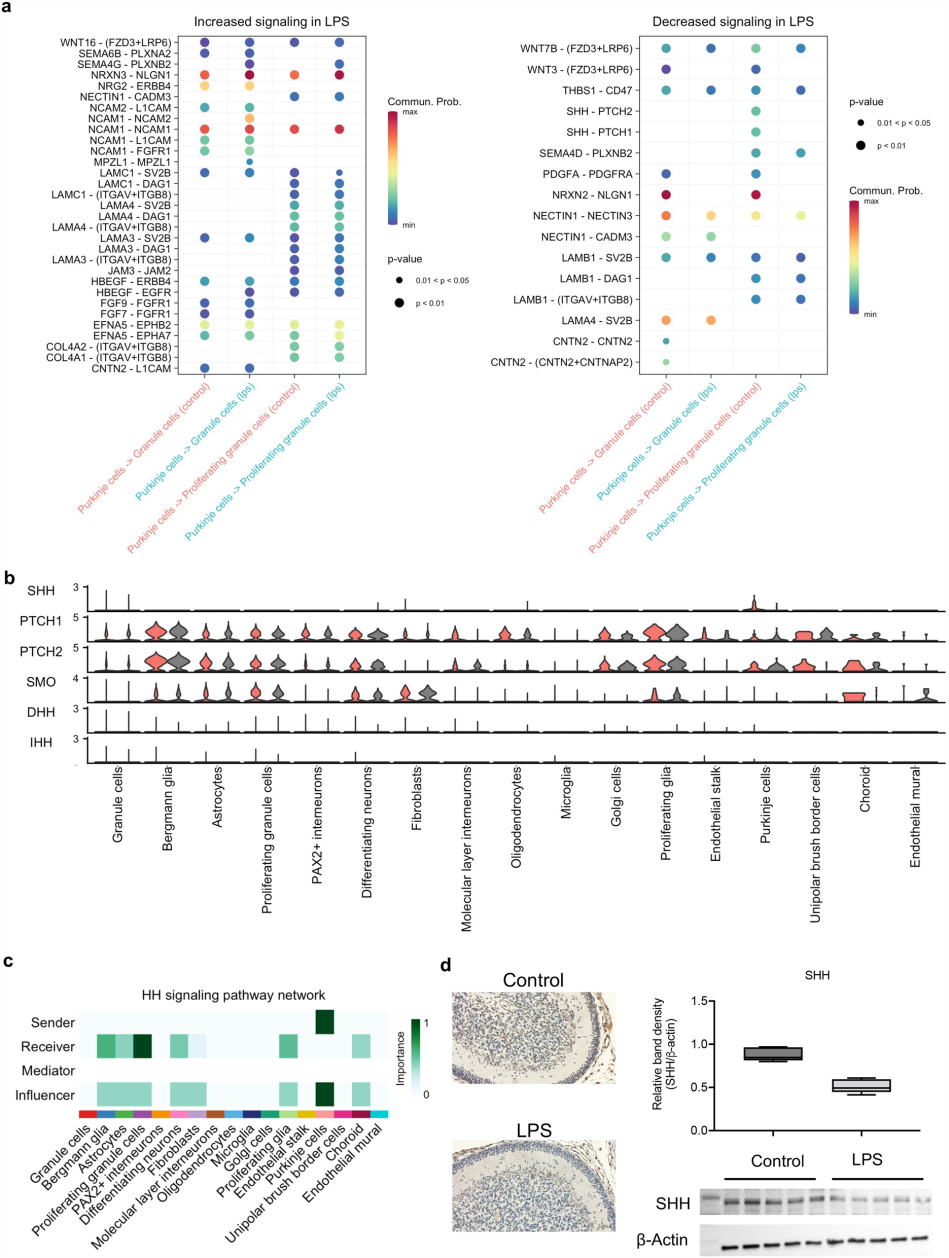
Chorioamnionitis impairs SHH signaling from Purkinje cells into proliferating GCPs/GCs. **a** Bubble plots of communication probability of ligand-receptors from Purkinje cells into GCPs and from Purkinje cells into GCs upregulated (right) and downregulated (left) in LPS compared to control. **b** Violin plot of signaling genes associated related to SHH signaling. **c** Heatmap of senders, receivers, mediators, and influencers of SHH signaling showing exclusive origin of SHH in Purkinje cells in controls and proliferating GCPs/GCs as the dominant receiver. **d** Immunohistochemistry and Western blot for SHH showing decreased SHH in the cerebellum of LPS-exposed fetuses compared to controls, *p* value < 0.001 (*n* = 5 animals/group)

Given the finding of suppressed HH signaling from Purkinje cells in LPS-exposed fetuses, we further analyzed the differential interactions from Purkinje cells to proliferating GCPs/GCs between LPS and control. We found several increased inferred interactions in LPS, such as NRG2–ERBB4 which is involved in the formation and maturation of synapses, neural cell adhesion molecule (NCAM) interactions that are involved in the final stages of axonal growth and synaptic stabilization, and FGF–FGFR interactions that inhibit SHH-mediated proliferation (Fig. 7a). Furthermore, we found decreased SHH-PTCH signaling in LPS, which is necessary for maintenance of GCP proliferation in the external granule layer (Fig. 7a). Analysis of the expression of the different molecules in the HH pathways revealed decreased expression of SHH in Purkinje cells (Fig. 7b). Detailed analysis of senders, receivers, and modulators of HH signaling in control animals showed Purkinje cells as the sole sender and proliferating GCPs as the major receiver. We then confirmed localization and expression of SHH at the protein level in the cerebellum (Fig. 7d). Immunohistochemistry showed SHH localized to Purkinje cell and GCPs/GCs with overall decreased signal in LPS. Western blot confirmed decreased SHH in LPS animals relative to control, corroborating that SHH is secreted by Purkinje cells and that SHH production and secretion of SHH is decreased in LPS (Fig. 7d).

### Chorioamnionitis promote maturation signaling in oligodendrocytes

**Fig. 8:**
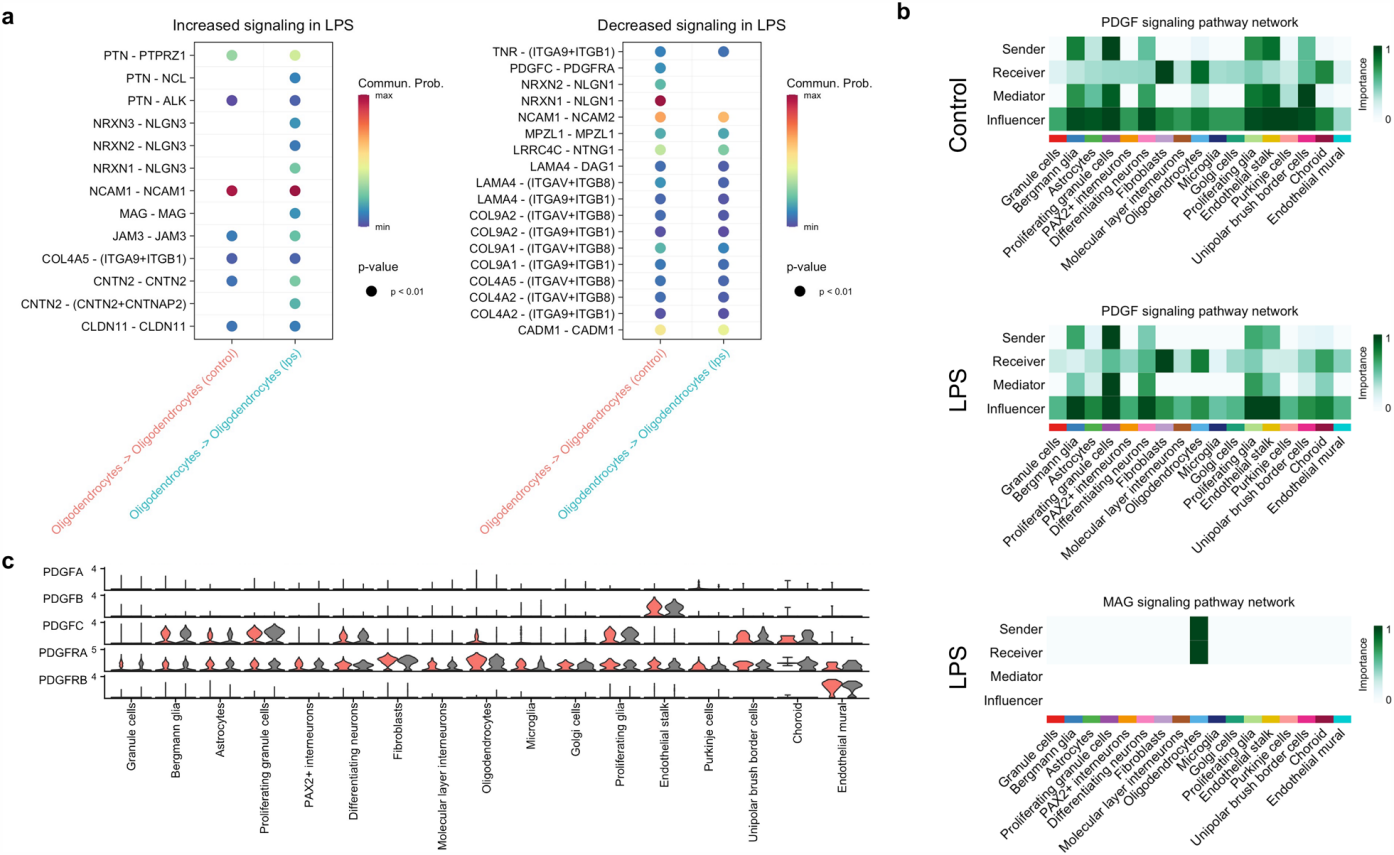
Chorioamnionitis impairs oligodendrocyte maturation signaling. **a** Bubble plots of communication probability of ligand-receptors from/to oligodendrocytes upregulated (right) and downregulated (left) by LPS compared to control. **b** Heatmap of senders, receivers, mediators, and influencers of PDGF and MAG signaling showing decreased PDGF signaling outgoing and incoming from oligodendrocytes in controls and exclusive MAG signaling in LPS. **c** Violin plot of signaling genes associated related to PDGF-PDGFRA signaling

We then delved into the signaling pathways involved in oligodendrocyte differentiation. During transition from oligodendrocyte precursor cell to myelinating oligodendrocyte there is decreased expression of PDGFRA and increased expression of myelin associated genes MBP, MAG, and myelin oligodendrocyte protein (MOG). We found that LPS increased MAG signaling and decreased PDGFC-PDGFRA in oligodendrocytes (Fig. 8a). Analysis of senders, receivers, and modulators showed that PDGF signaling from proliferating glia, endothelial stalk cells, and UBC was decreased in LPS compared to controls; while MAG signaling was exclusive to LPS-exposed fetuses (Fig. 8b). At the gene level, chorioamnionitis decreased the expression of PDGFC and PDGFRA and increased the expression of MBP, MAG, and MOG (Fig. 8c), consistent with our finding of decreased MBP at the protein level (Fig. 4d).

## Discussion

In this study we demonstrate, in a clinically relevant model of prematurity and chorioamnionitis using preterm Rhesus macaques, that antenatal inflammation disrupts cerebellar development by impairing hedgehog signaling from Purkinje cells. Impaired hedgehog signaling was associated with accelerated maturation of GCss and oligodendrocytes on single-nuclei analyses confirmed by translational analyses we show that chorioamnionitis leads to a decrease in the number of Purkinje cells with accelerated maturation of GCss and oligodendrocytes in the developing cerebellum. We also determine that decreased Purkinje cell-derived SHH signaling to GCs and pre-oligodendrocytes is driving the disrupted cerebellar development. These findings are consistent with autopsy of individuals with ASD [10].

The improved survival of extreme preterm infants is obviating the association of preterm birth and ASD. A large cohort study of over 4 million singleton births showed that prevalence of autism in extremely preterm births was as high as 6.1% [40]. A recent metanalysis including 18 studies examining the prevalence of ASD in preterm infants found a similar prevalence rate of 7% [2]. Development of ASD is also linked to prenatal inflammation. The presence of histological chorioamnionitis carries an increased risk of development of ASD [4]. Follow-up from a large cohort of preterm infants showed an increased risk for ASD when histological chorioamnionitis was present [41]. The clinical link between antenatal inflammation and neurodevelopmental disorders with ASD-like phenotype is corroborated by animal studies. In mice, maternal immune activation disrupts the integrated stress response in the fetal brain leading to neurobehavioral abnormalities [15]. In pregnant rats injected with intraperitoneal Group B Streptococcus there was histological evidence of chorioamnionitis and the offspring was more likely to show abnormal social interaction, impaired communication, and hyperactivity [42].

In the preterm rhesus macaque model IA LPS has been shown to induce robust cell neutrophilic recruitment and the chorio-decidual interface, which is associated with systemic fetal inflammatory response [20, 38]. In this model, we have observed early neuroinflammation of the cerebellum with increased expression of cytokines and increased IL-6 in the CSF [37]. At 5 days we found a more subtle but persistent neutrophilic infiltrate and inflammation of the amniotic membranes. The fetal systemic inflammation induced by IA LPS was associated with decreased number of Purkinje cells by snRNA-seq and a trend of decreased density of Purkinje cells on histology. Loss of cerebellar Purkinje cells is among the most observed histopathological findings in autism [10, 43, 44]. Experimental data from Lurcher mice, which are genetically programmed to have variable degrees of Purkinje cell death after birth, show a correlation between loss of Purkinje cells and deficits on serial reversal-learning task, which measures low order behavioral flexibility [43]. The mechanisms of Purkinje cell loss need to be further explored at earlier timepoints.

Purkinje cells are master regulators of cerebellar development [45]. In rodents, there is a critical window of cerebellar development during the first 2 weeks of life which corresponds to third trimester cerebellar development in primates. During this period, different perinatal insults can lead to Purkinje cell loss and dysfunction. In a rodent model of neonatal brain injury induced by hypoxia, there is delayed Purkinje cell arborization and reduction in firing associated with long-term cerebellar learning deficits that can be partially restored by GABA reuptake inhibitors [46, 47]. Exposure to systemic LPS in the second week of life of rodents induces prostaglandin E2 production in the cerebellum and administration of either LPS or prostaglandin E2 at this impairs growth of the Purkinje cell dendritic tree and reduces social play behavior in males, suggesting a developmental-specific role of prostaglandins in cerebellar injury. In a similar model, IA LPS increases cerebellar COX-2 mRNA in the cerebellum of rhesus macaques at 130 days of gestation [37]. Moreover, genes that regulate Purkinje cell development have been implicated in the pathogenesis of ASD. The autism susceptibility candidate 2 (AUTS2) was identified as a risk gene for ASD in human genetic studies [48]. Interestingly, AUTS2 is selectively expressed in Purkinje cells and Golgi cells during postnatal development in rodents and conditional deletion of AUTS2 results in an underdeveloped cerebellum with immature Purkinje cells and impaired motor learning and vocal communication [49].

Purkinje cells regulate cerebellar GC maturation by maintaining their proliferative status through SHH signaling [50]. We found that chorioamnionitis led to decreased SHH signaling in Purkinje cells and accelerated maturation of GCs, with decreased SHH expression in chorioamnionitis confirmed at the protein level. A recent study of preterm baboons without exposure to prenatal inflammation showed that preterm birth was associated with structural and functional changes in Purkinje cells, including decreased synaptic input and abnormal action potentials firing and adaptation [51]. Autopsy of preterm infants showed decreased thickness of the internal and external granule layers with decreased expression of SHH in the Purkinje cell layer compared to term stillborn infants [19]. Our findings show that prenatal inflammation leads to Purkinje cell loss and disrupted development of GCs in the developing cerebellum of preterm fetuses that may have important implications on long-term neurodevelopmental outcomes. In the changes we observed, in addition to disrupted neuronal maturation, we found that chorioamnionitis also accelerated pre-oligodendrocyte maturation into myelinating oligodendrocytes, evidenced by an increase in myelin-associated genes including MBP and MAG. This finding was consistent with increased expression of MBP on protein analysis and appears to be unique to the cerebellum. A postmortem study from patients with autism showed that in the cerebral white matter myelin-related proteins are decreased, but in the cerebellum they are increased [52]. In addition, a mouse model of placental endocrine deficiency showed sex-specific effects in cerebellar myelination, with increased myelin in male, which was associated with neurobehavioral abnormalities consistent with autism [53]. While SHH signaling is not a classic mechanism of oligodendrocyte maturation, in vitro studies with cerebellar organotypic cultures have shown that SHH produced by Purkinje cells stimulates the proliferation of oligodendrocyte precursor cells and the decreased SHH production during postnatal development is associated with maturation of oligodendrocytes in the cerebellum [53]. This data suggests that the loss of Purkinje cells with decreased SHH signaling in LPS-exposed fetuses may be a common mechanism to accelerated GC and oligodendrocyte maturation.

While our study relied primarily on bioinformatics, which was limited by the number of animals used in the analyses, we used histopathological assessment to confirm our findings in a larger number of animals. Despite this limitation, the use of a nonhuman primate model provides highly translational insight into the effects of antenatal inflammation on cerebellar development. We also were not able to assess the cause of Purkinje cell loss, which would require additional studies at earlier timepoint to assess for mechanisms of cell injury and death as these cells are already differentiated at the time of the insult. In addition, we were not able to confirm histologically the increase in UBCs identified on single cell. Finally, postnatal behavioral studies would also provide additional insight into the functional significance of our findings.

## Conclusions

Overall, our findings in a preterm nonhuman primate model of chorioamnionitis support the role of prenatal inflammation in disrupted cerebellar development through reduction of Purkinje cells associated with accelerated maturation of GCs and oligodendrocytes. The link between preterm birth and development of ASD is well-established, but the specific mechanisms are unclear. Our results suggest that prenatal inflammation, a common cause of preterm birth, contributes to disrupted cerebellar development leading to changes like the histopathological findings of ASD in the cerebellum. The mechanisms of chorioamnionitis leading to disrupted cerebellar development and ASD may be common to other inflammatory exposures during pregnancy including congenital infections and viral illnesses such as influenza.

### Supplementary Information

**Additional file 1: Fig. S1.** Flow cytometry of the chorio-decidua cells.

**Additional file 2: Fig. S2.** Multiplex ELISA for cytokines in the maternal and fetal plasma.

**Additional file 3: Fig. S3.** Microglial diversity in the developing cerebellum. **a,** UMAP plot of the microglia cluster showing 3 subpopulations. **b,** Heatmap of top differentially expressed genes in each microglia cluster **c,** UMAP plot of microglia cluster by condition. **d,** Expression of canonical and cluster-specific microglial markers. **e**, Gene set enrichment analysis for Biological processes of genes differentially regulated in cluster 0. **f**, Gene set enrichment analysis for Biological processes of genes differentially regulated in cluster 1. **g**, Gene set enrichment analysis for Biological processes of genes differentially regulated in cluster 2.

**Additional file 4: Fig. S4.** Astrocyte diversity in the developing cerebellum. **a,** UMAP plot of the astrocyte cluster showing 3 subpopulations. **b,** Heatmap of top differentially expressed genes in each astrocyte cluster **c,** UMAP plot of astrocyte clusters by condition. **d,** Expression of cluster-specific markers. **e**, Gene set enrichment analysis for Biological processes of genes differentially regulated in cluster 0. **f**, Gene set enrichment analysis for Biological processes of genes differentially regulated in cluster 1. **g**, Gene set enrichment analysis for Biological processes of genes differentially regulated in cluster 2. **h**, Gene set enrichment analysis for Biological processes of genes differentially regulated in cluster 3.

**Additional file 5: Fig. S5.** Classification of cellular communication networks based on function similarity.

**Additional file 6: Table S1.** Top 10 differentially expressed genes in each of the 24 clusters identified, used for cell type identification.

**Additional file 7: Table S2.** Top 10 differentially expressed genes for specific cell clusters: GCPs/GCs, Purkinje cells, oligodendrocytes, microglia, and astrocytes.

**Additional file 8: Table S3.** Differentially expressed genes in LPS compared to controls in the GCPs/GCs clusters.

**Additional file 9: Table S4.** Differentially expressed genes in LPS compared to controls in the Purkinje cell cluster.

**Additional file 10: Table S5.** Differentially expressed genes in LPS compared to controls in the oligodendrocyte cluster.

## Data Availability

The gene expression data have been deposited in NCBI’s Gene Expression Omnibus (GEO) and are accessible through GEO Series accession no. GSE205001.

## Abbreviations

ASD: Autism spectrum disorder
BMP: Bone morphogenetic protein
CADM2: Cell adhesion molecule 2
CX3CR1: C-X3-C motif chemokine receptor
DAB2: Disable 2
EGL: External granule layer
EPHB: EphrinB
FGF: Fibroblast growth factor
FGFR: Fibroblast growth factor receptor
GC: Granule cell
GCP: Granule cell precursor
HH: Hedgehog
IA: Intra-amniotic
IGL: Internal granule layer
IL-6: Interleukin-6
IL-17: Interleukin-17
IL-1ra: Interleukin-1 receptor antagonist protein
LPS: Lipopolysaccharide
MAG: Myelin-associated glycoprotein
MBP: Myelin basic protein
NCAM: Neural cell adhesion molecule
NGL: Netrin-F ligand
OPC: Oligodendrocyte progenitor cell
PCA: Principal component analysis
PDGFC: Platelet-derived growth factor C
PDGFRA: Platelet-derived growth factor receptor α
PLXDC2: Plexin domain containing 2
PTCH: Patched
SHH: Sonic Hedgehog
snRNA-seq: Single-nuclei RNA sequencing
TOP2A: Topoisomerase IIα
TREM2: Triggering receptor expressed on myeloid cells 2
UBC: Unipolar brush border cell
WNT: Wingless int

## References

[1] Maenner MJ, Shaw KA, Bakian AV, Bilder DA, Durkin MS, Esler A (2021). Prevalence and characteristics of autism spectrum disorder among children aged 8 years—autism and developmental disabilities monitoring network, 11 sites, United States, 2018. MMWR Surveill Summ.

[2] Agrawal S, Rao SC, Bulsara MK, Patole SK (2018). Prevalence of autism spectrum disorder in preterm infants: a meta-analysis. Pediatrics.

[3] Romero R, Miranda J, Chaiworapongsa T, Korzeniewski SJ, Chaemsaithong P, Gotsch F (2014). Prevalence and clinical significance of sterile intra-amniotic inflammation in patients with preterm labor and intact membranes. Am J Reprod Immunol.

[4] Tsamantioti E, Lisonkova S, Muraca G, Ortqvist AK, Razaz N (2022). Chorioamnionitis and risk of long-term neurodevelopmental disorders in offspring: a population-based cohort study. Am J Obstet Gynecol.

[5] Straughen JK, Misra DP, Divine G, Shah R, Perez G, VanHorn S (2017). The association between placental histopathology and autism spectrum disorder. Placenta.

[6] Volpe JJ (2021). Commentary—Cerebellar underdevelopment in the very preterm infant: important and underestimated source of cognitive deficits. J Neonatal Perinatal Med.

[7] Limperopoulos C, Bassan H, Gauvreau K, Robertson RL, Sullivan NR, Benson CB (2007). Does cerebellar injury in premature infants contribute to the high prevalence of long-term cognitive, learning, and behavioral disability in survivors?. Pediatrics.

[8] Schmahmann JD (2004). Disorders of the cerebellum: ataxia, dysmetria of thought, and the cerebellar cognitive affective syndrome. J Neuropsychiatry Clin Neurosci.

[9] Sathyanesan A, Zhou J, Scafidi J, Heck DH, Sillitoe RV, Gallo V (2019). Emerging connections between cerebellar development, behaviour and complex brain disorders. Nat Rev Neurosci.

[10] Fatemi SH, Aldinger KA, Ashwood P, Bauman ML, Blaha CD, Blatt GJ (2012). Consensus paper: pathological role of the cerebellum in autism. Cerebellum.

[11] Butts T, Green MJ, Wingate RJ (2014). Development of the cerebellum: simple steps to make a 'little brain'. Development.

[12] Matthews LG, Inder TE, Pascoe L, Kapur K, Lee KJ, Monson BB (2018). Longitudinal preterm cerebellar volume: perinatal and neurodevelopmental outcome associations. Cerebellum.

[13] Parker J, Mitchell A, Kalpakidou A, Walshe M, Jung HY, Nosarti C (2008). Cerebellar growth and behavioural & neuropsychological outcome in preterm adolescents. Brain.

[14] Allin M, Matsumoto H, Santhouse AM, Nosarti C, AlAsady MH, Stewart AL (2001). Cognitive and motor function and the size of the cerebellum in adolescents born very pre-term. Brain.

[15] Kalish BT, Kim E, Finander B, Duffy EE, Kim H, Gilman CK (2021). Maternal immune activation in mice disrupts proteostasis in the fetal brain. Nat Neurosci.

[16] Volpe JJ (2009). Brain injury in premature infants: a complex amalgam of destructive and developmental disturbances. Lancet Neurol.

[17] Pierrat V, Marchand-Martin L, Arnaud C, Kaminski M, Resche-Rigon M, Lebeaux C (2017). Neurodevelopmental outcome at 2 years for preterm children born at 22 to 34 weeks’ gestation in France in 2011: EPIPAGE-2 cohort study. BMJ Brit Med J..

[18] Courchesne E, Karns CM, Davis HR, Ziccardi R, Carper RA, Tigue ZD (2001). Unusual brain growth patterns in early life in patients with autistic disorder: an MRI study. Neurology.

[19] Haldipur P, Bharti U, Alberti C, Sarkar C, Gulati G, Iyengar S (2011). Preterm delivery disrupts the developmental program of the cerebellum. PLoS ONE.

[20] Presicce P, Park CW, Senthamaraikannan P, Bhattacharyya S, Jackson C, Kong F, et al. IL-1 signaling mediates intrauterine inflammation and chorio-decidua neutrophil recruitment and activation. JCI Insight. 2018;3(6). 10.1172/jci.insight.98306 PMC5926925 29563340

[21] Stuart T, Butler A, Hoffman P, Hafemeister C, Papalexi E, Mauck WM (2019). Comprehensive integration of single-cell data. Cell.

[22] Young MDB, S. SoupX removes ambient RNA contamination from droplet-based single-cell RNA sequencing data. GigaScience. 2020. 10.1093/gigascience/giaa151 PMC7763177 33367645

[23] Kozareva V, Martin C, Osorno T, Rudolph S, Guo C, Vanderburg C (2021). A transcriptomic atlas of mouse cerebellar cortex comprehensively defines cell types. Nature.

[24] Wizeman JW, Guo Q, Wilion EM, Li JY. Specification of diverse cell types during early neurogenesis of the mouse cerebellum. Elife. 2019;8. 10.7554/eLife.42388 PMC6382353 30735127

[25] Carter RA, Bihannic L, Rosencrance C, Hadley JL, Tong Y, Phoenix TN (2018). A single-cell transcriptional atlas of the developing murine cerebellum. Curr Biol.

[26] Single Cell Proportion Test [Internet]. 2020. Available from: https://github.com/rpolicastro/scProportionTest.

[27] Kaimal V, Bardes EE, Tabar SC, Jegga AG, Aronow BJ (2010). ToppCluster: a multiple gene list feature analyzer for comparative enrichment clustering and network-based dissection of biological systems. Nucleic Acids Res.

[28] Xie Z, Bailey A, Kuleshov MV, Clarke DJB, Evangelista JE, Jenkins SL (2021). Gene set knowledge discovery with enrichr. Curr Protoc.

[29] Kuleshov MV, Jones MR, Rouillard AD, Fernandez NF, Duan Q, Wang Z (2016). Enrichr: a comprehensive gene set enrichment analysis web server 2016 update. Nucleic Acids Res.

[30] Trapnell C, Cacchiarelli D, Grimsby J, Pokharel P, Li S, Morse M (2014). The dynamics and regulators of cell fate decisions are revealed by pseudotemporal ordering of single cells. Nat Biotechnol.

[31] Qiu X, Mao Q, Tang Y, Wang L, Chawla R, Pliner HA (2017). Reversed graph embedding resolves complex single-cell trajectories. Nat Methods.

[32] Cao J, Spielmann M, Qiu X, Huang X, Ibrahim DM, Hill AJ (2019). The single-cell transcriptional landscape of mammalian organogenesis. Nature.

[33] Street K, Risso D, Fletcher RB, Das D, Ngai J, Yosef N (2018). Slingshot: cell lineage and pseudotime inference for single-cell transcriptomics. BMC Genomics.

[34] de Bézieux HR, Van den Berge K, Street K, Dudoit S (2021). Trajectory inference across multiple conditions with condiments: differential topology, progression, differentiation, and expression. BioRxiv..

[35] McInnes LH, J.; Melville, J. UMAP: uniform manifold approximation and projection for dimension reduction 2018. Available from: https://arxiv.org/abs/1802.03426.

[36] Jin S, Guerrero-Juarez CF, Zhang L, Chang I, Ramos R, Kuan CH (2021). Inference and analysis of cell–cell communication using Cell Chat. Nat Commun.

[37] Schmidt AF, Kannan PS, Chougnet CA, Danzer SC, Miller LA, Jobe AH (2016). Intra-amniotic LPS causes acute neuroinflammation in preterm rhesus macaques. J Neuroinflammation.

[38] Rueda CM, Presicce P, Jackson CM, Miller LA, Kallapur SG, Jobe AH (2016). Lipopolysaccharide-induced chorioamnionitis promotes IL-1-dependent inflammatory FOXP3+ CD4+ T cells in the fetal rhesus macaque. J Immunol.

[39] Louis ED, Babij R, Lee M, Cortes E, Vonsattel JP (2013). Quantification of cerebellar hemispheric Purkinje cell linear density: 32 ET cases versus 16 controls. Mov Disord.

[40] Bokobza C, Van Steenwinckel J, Mani S, Mezger V, Fleiss B, Gressens P (2019). Neuroinflammation in preterm babies and autism spectrum disorders. Pediatr Res.

[41] Venkatesh KK, Leviton A, Hecht JL, Joseph RM, Douglass LM, Frazier JA (2020). Histologic chorioamnionitis and risk of neurodevelopmental impairment at age 10 years among extremely preterm infants born before 28 weeks of gestation. Am J Obstet Gynecol.

[42] Allard MJ, Bergeron JD, Baharnoori M, Srivastava LK, Fortier LC, Poyart C (2017). A sexually dichotomous, autistic-like phenotype is induced by Group B Streptococcus maternofetal immune activation. Autism Res.

[43] Cairns J, Swanson D, Yeung J, Sinova A, Chan R, Potluri P (2017). Abnormalities in the structure and function of cerebellar neurons and neuroglia in the Lc/+ chimeric mouse model of variable developmental Purkinje cell loss. Cerebellum.

[44] Rahi S, Mehan S (2022). Understanding abnormal SMO-SHH signaling in autism spectrum disorder: potential drug target and therapeutic goals. Cell Mol Neurobiol.

[45] Fleming J, Chiang C (2015). The Purkinje neuron: a central orchestrator of cerebellar neurogenesis. Neurogenesis (Austin).

[46] Sathyanesan A, Kundu S, Abbah J, Gallo V (2018). Neonatal brain injury causes cerebellar learning deficits and Purkinje cell dysfunction. Nat Commun.

[47] Zonouzi M, Scafidi J, Li P, McEllin B, Edwards J, Dupree JL (2015). GABAergic regulation of cerebellar NG2 cell development is altered in perinatal white matter injury. Nat Neurosci.

[48] Oksenberg N, Ahituv N (2013). The role of AUTS2 in neurodevelopment and human evolution. Trends Genet.

[49] Yamashiro K, Hori K, Lai ESK, Aoki R, Shimaoka K, Arimura N (2020). AUTS2 governs cerebellar development, Purkinje cell maturation, motor function and social communication. iScience.

[50] Lewis PM, Gritli-Linde A, Smeyne R, Kottmann A, McMahon AP (2004). Sonic hedgehog signaling is required for expansion of granule neuron precursors and patterning of the mouse cerebellum. Dev Biol.

[51] Barron T, Kim JH (2020). Preterm birth impedes structural and functional development of cerebellar Purkinje cells in the developing baboon cerebellum. Brain Sci.

[52] Broek JA, Guest PC, Rahmoune H, Bahn S (2014). Proteomic analysis of post mortem brain tissue from autism patients: evidence for opposite changes in prefrontal cortex and cerebellum in synaptic connectivity-related proteins. Mol Autism.

[53] Vacher CM, Lacaille H, O'Reilly JJ, Salzbank J, Bakalar D, Sebaoui S (2021). Placental endocrine function shapes cerebellar development and social behavior. Nat Neurosci.

