# Supplementary figures and images for: Chorioamnionitis accelerates granule cell and oligodendrocyte maturation in the cerebellum of preterm nonhuman primates
Source: J Neuroinflammation. 2024 Jan 10;21:16. doi: 10.1186/s12974-024-03012-y (PMC10777625; doi:10.1186/s12974-024-03012-y)

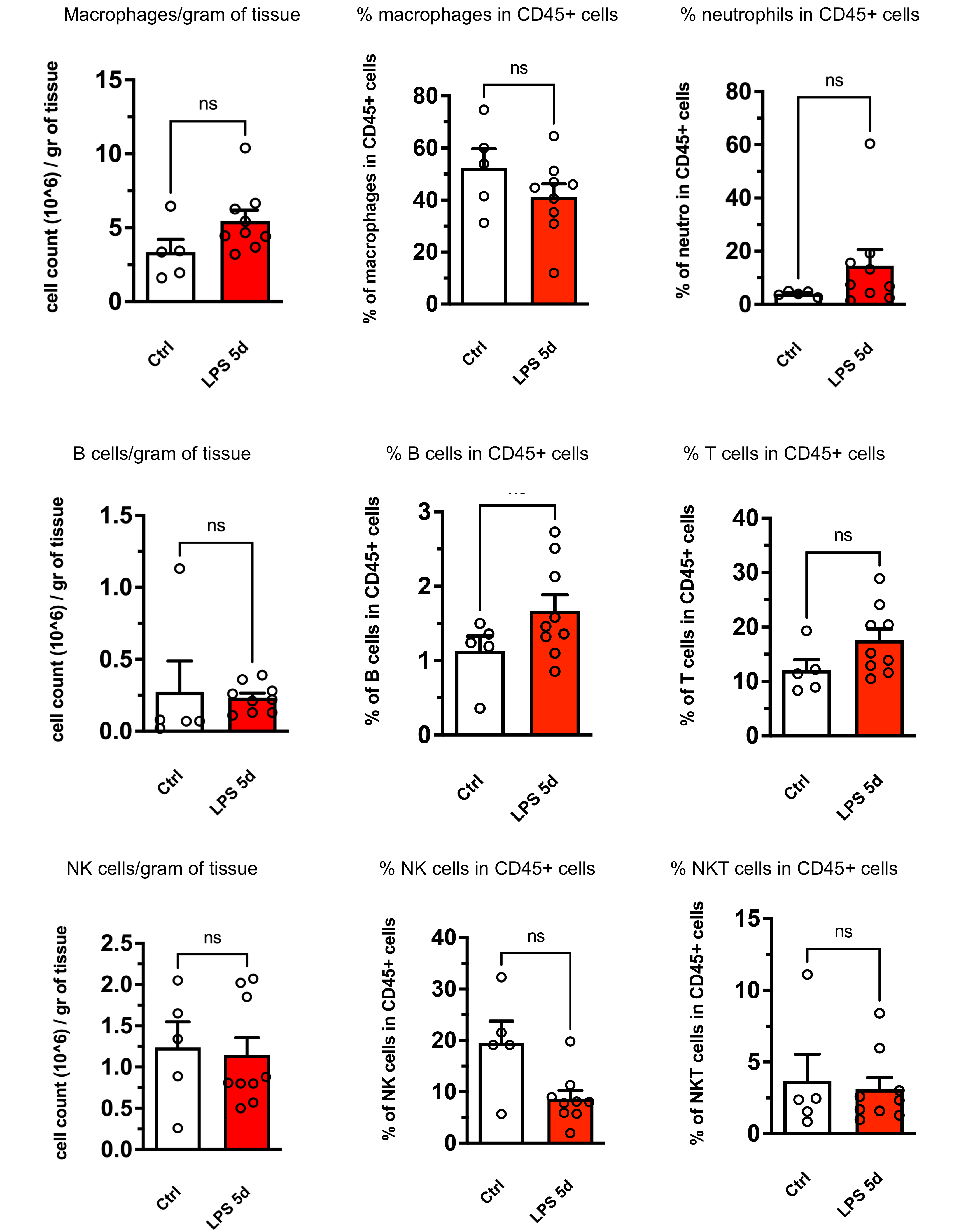

Supplement: Supplementary file 1 — Additional file 1: Fig. S1. Flow cytometry of the chorio-decidua cells. [file 12974_2024_3012_MOESM1_ESM.tif]

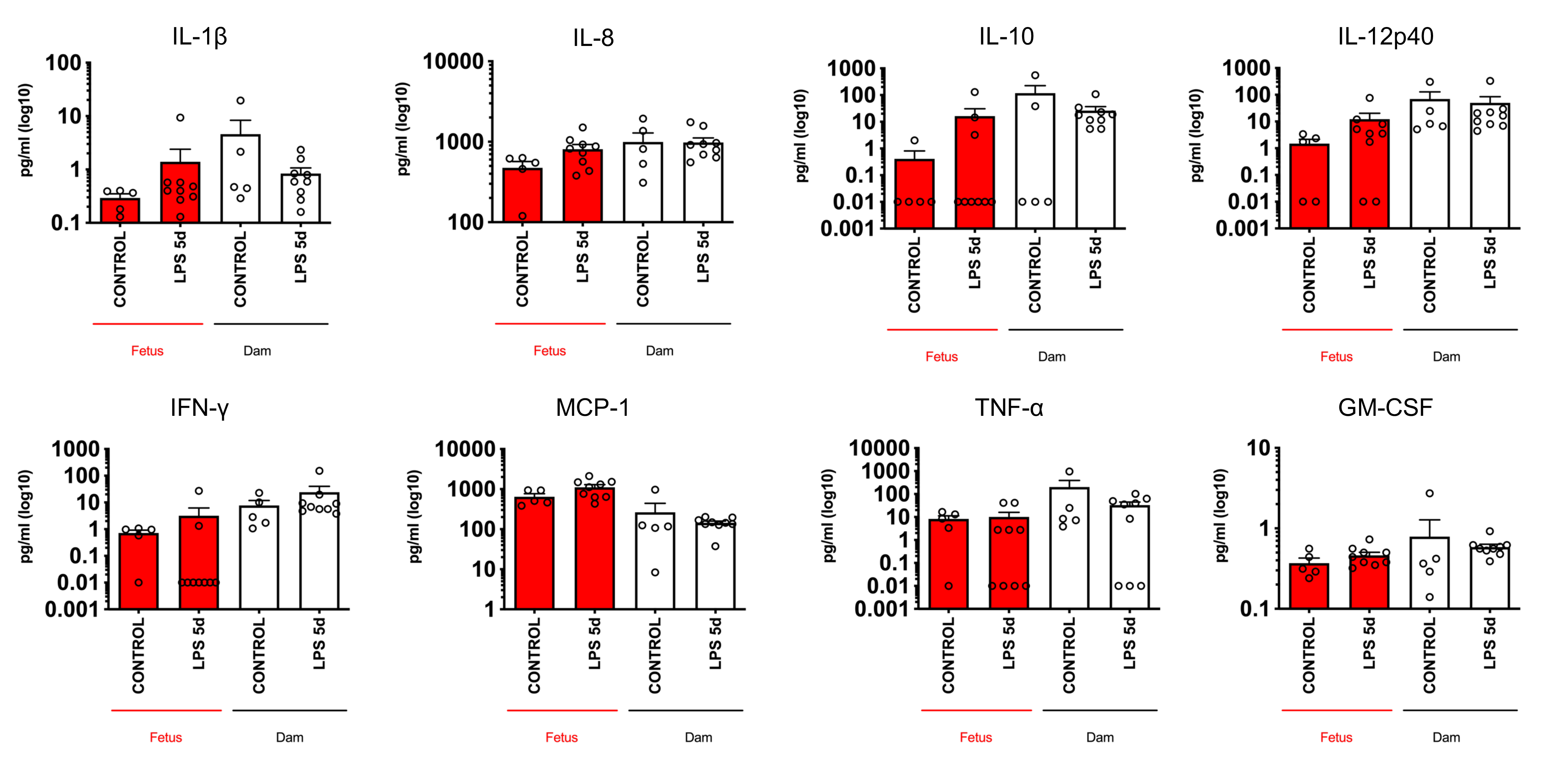

Supplement: Supplementary file 2 — Additional file 2: Fig. S2. Multiplex ELISA for cytokines in the maternal and fetal plasma. [file 12974_2024_3012_MOESM2_ESM.tif]

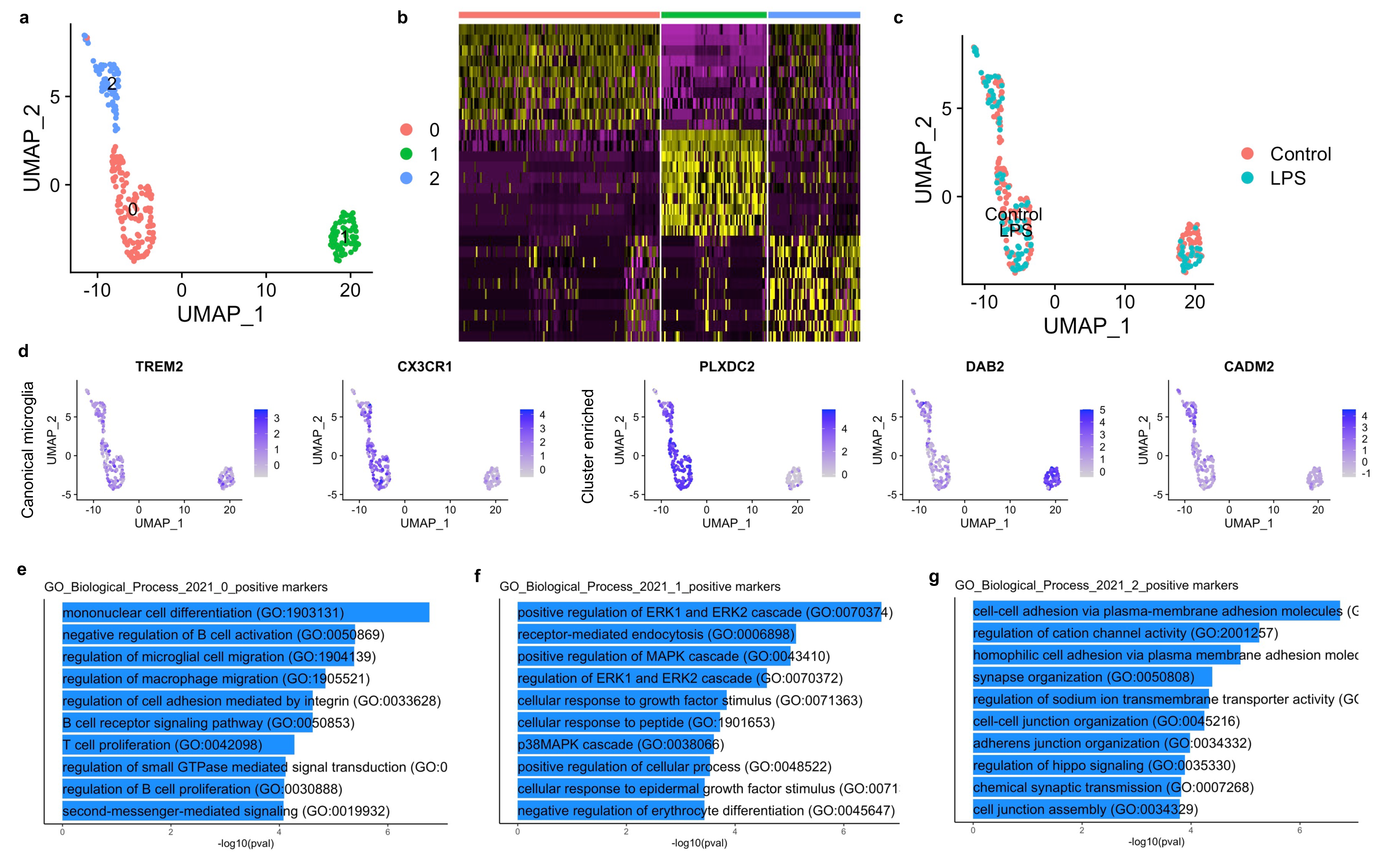

Supplement: Supplementary file 3 — Additional file 3: Fig. S3. Microglial diversity in the developing cerebellum. a, UMAP plot of the microglia cluster showing 3 subpopulations. b, Heatmap of top differentially expressed genes in each microglia cluster c, UMAP plot of microglia cluster by condition. d, Expression of canonical and cluster-specific microglial markers. e, Gene set enrichment analysis for Biological processes of genes differentially regulated in cluster 0. f, Gene set enrichment analysis for Biological processes of genes differentially regulated in cluster 1. g, Gene set enrichment analysis for Biological processes of genes differentially regulated in cluster 2. [file 12974_2024_3012_MOESM3_ESM.tif]

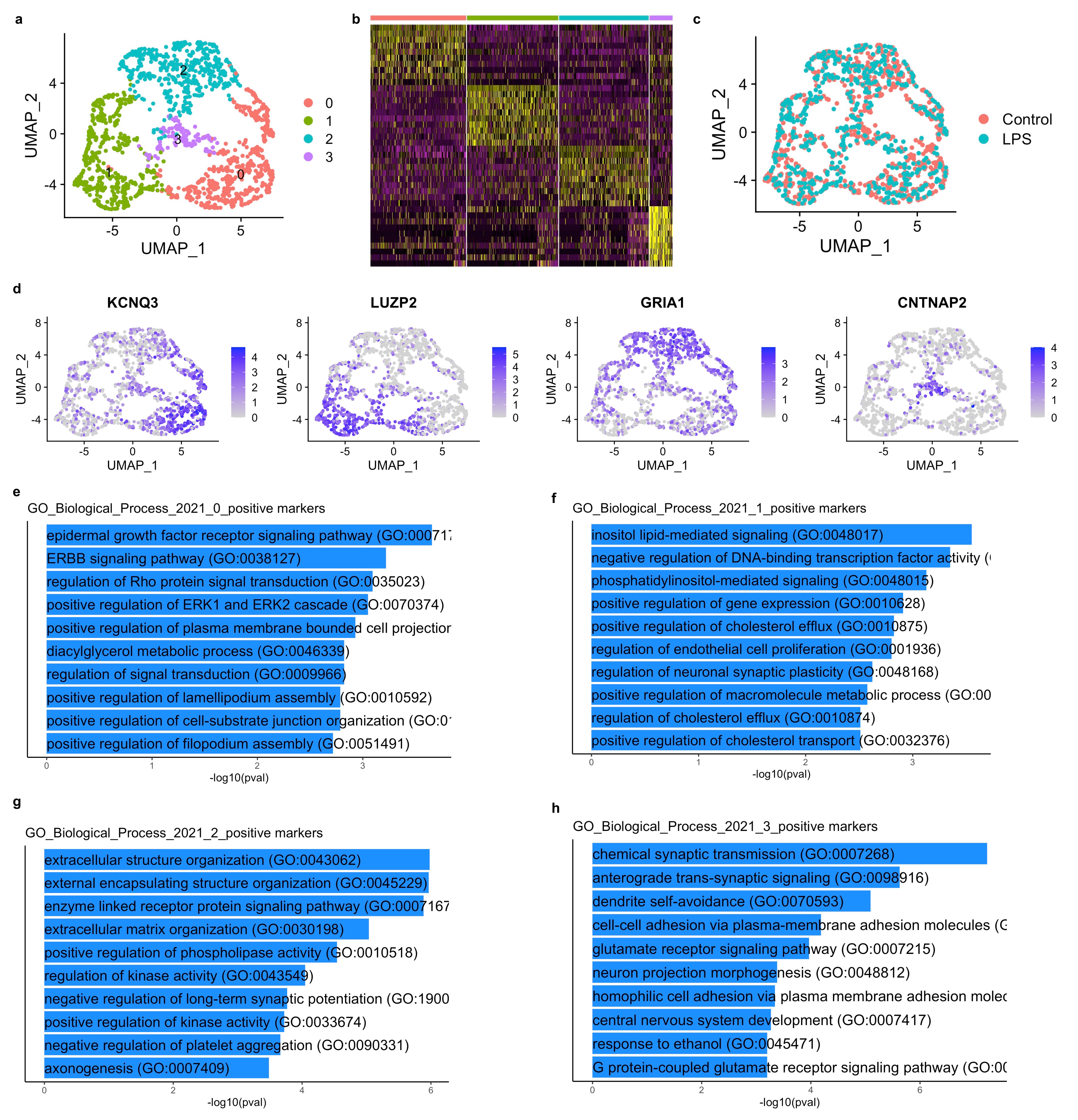

Supplement: Supplementary file 4 — Additional file 4: Fig. S4. Astrocyte diversity in the developing cerebellum. a, UMAP plot of the astrocyte cluster showing 3 subpopulations. b, Heatmap of top differentially expressed genes in each astrocyte cluster c, UMAP plot of astrocyte clusters by condition. d, Expression of cluster-specific markers. e, Gene set enrichment analysis for Biological processes of genes differentially regulated in cluster 0. f, Gene set enrichment analysis for Biological processes of genes differentially regulated in cluster 1. g, Gene set enrichment analysis for Biological processes of genes differentially regulated in cluster 2. h, Gene set enrichment analysis for Biological processes of genes differentially regulated in cluster 3. [file 12974_2024_3012_MOESM4_ESM.tif]

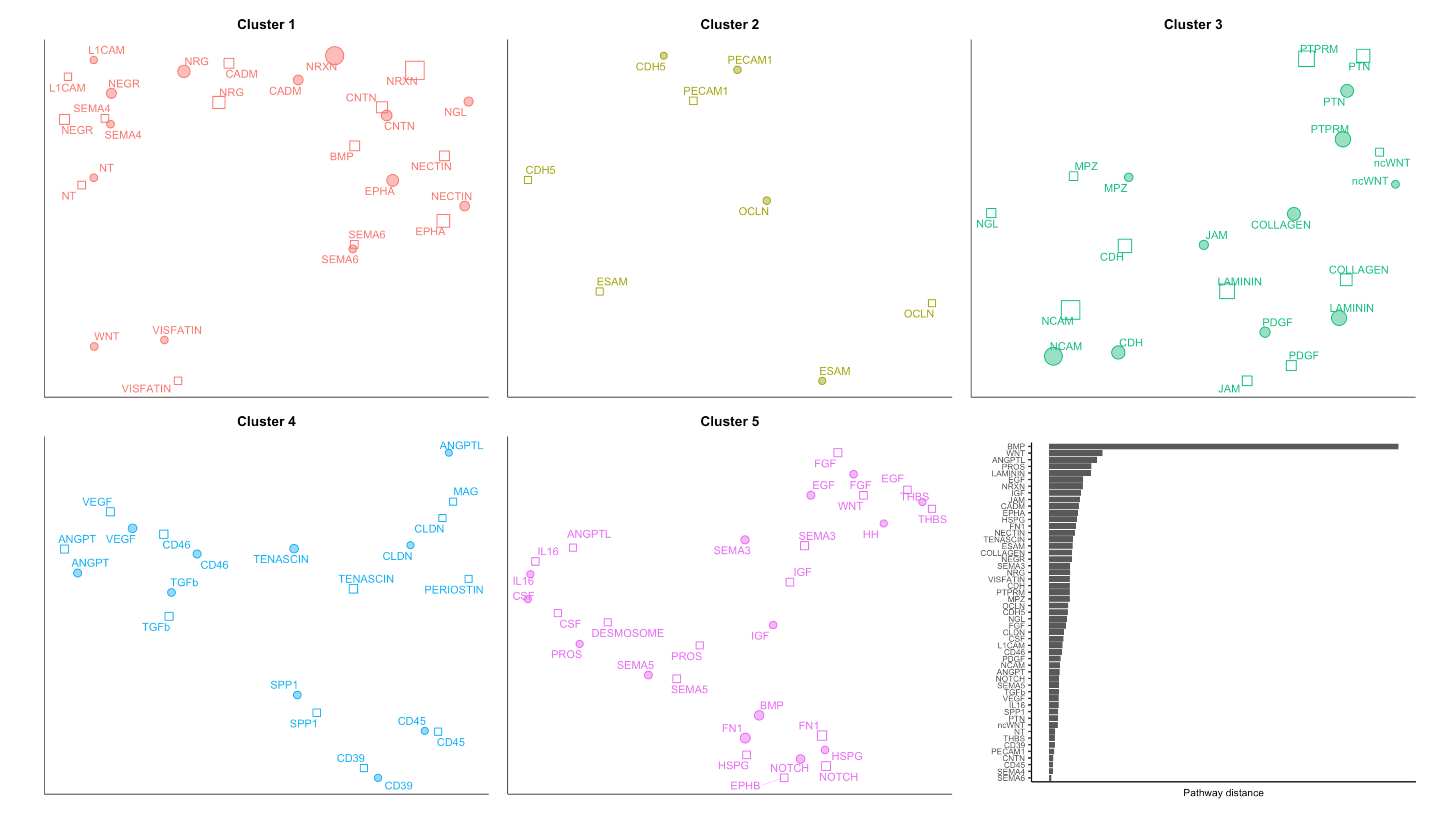

Supplement: Supplementary file 5 — Additional file 5: Fig. S5. Classification of cellular communication networks based on function similarity. [file 12974_2024_3012_MOESM5_ESM.tif]
